# Huntington’s disease affects mitochondrial network dynamics predisposing to pathogenic mitochondrial DNA mutations

**DOI:** 10.1093/brain/awae007

**Published:** 2024-01-09

**Authors:** Andreas Neueder, Kerstin Kojer, Zhenglong Gu, Yiqin Wang, Tanja Hering, Sarah Tabrizi, Jan-Willem Taanman, Michael Orth

**Affiliations:** Department of Neurology, Ulm University, 89081 Ulm, Germany; Department of Neurology, Ulm University, 89081 Ulm, Germany; Division of Nutritional Sciences, Cornell University, Ithaca, NY 14853, USA; Division of Nutritional Sciences, Cornell University, Ithaca, NY 14853, USA; Department of Neurology, Ulm University, 89081 Ulm, Germany; UCL Huntington’s Disease Centre, UCL Queen Square Institute of Neurology and National Hospital for Neurology and Neurosurgery, London WC1N 3BG, UK; Dementia Research Institute at UCL, London WC1N 3BG, UK; Department of Clinical and Movement Neurosciences, UCL Queen Square Institute of Neurology, London NW3 2PF, UK; Department of Neurology, Ulm University, 89081 Ulm, Germany; Swiss Huntington Centre, Siloah AG, 3073 Gümligen, Switzerland; University Hospital of Old Age Psychiatry and Psychotherapy, Bern University, CH-3000 Bern 60, Switzerland

**Keywords:** mitochondrial fission, mitophagy, huntingtin fragments, DNA instability, ultra-deep mitochondrial DNA sequencing, proteomics

## Abstract

Huntington’s disease (HD) predominantly affects the brain, causing a mixed movement disorder, cognitive decline and behavioural abnormalities. It also causes a peripheral phenotype involving skeletal muscle. Mitochondrial dysfunction has been reported in tissues of HD models, including skeletal muscle, and lymphoblast and fibroblast cultures from patients with HD. Mutant huntingtin protein (mutHTT) expression can impair mitochondrial quality control and accelerate mitochondrial ageing.

Here, we obtained fresh human skeletal muscle, a post-mitotic tissue expressing the mutated HTT allele at physiological levels since birth, and primary cell lines from HTT CAG repeat expansion mutation carriers and matched healthy volunteers to examine whether such a mitochondrial phenotype exists in human HD. Using ultra-deep mitochondrial DNA (mtDNA) sequencing, we showed an accumulation of mtDNA mutations affecting oxidative phosphorylation. Tissue proteomics indicated impairments in mtDNA maintenance with increased mitochondrial biogenesis of less efficient oxidative phosphorylation (lower complex I and IV activity). In full-length mutHTT expressing primary human cell lines, fission-inducing mitochondrial stress resulted in normal mitophagy. In contrast, expression of high levels of N-terminal mutHTT fragments promoted mitochondrial fission and resulted in slower, less dynamic mitophagy. Expression of high levels of mutHTT fragments due to somatic nuclear HTT CAG instability can thus affect mitochondrial network dynamics and mitophagy, leading to pathogenic mtDNA mutations.

We show that life-long expression of mutant HTT causes a mitochondrial phenotype indicative of mtDNA instability in fresh post-mitotic human skeletal muscle. Thus, genomic instability may not be limited to nuclear DNA, where it results in somatic expansion of the HTT CAG repeat length in particularly vulnerable cells such as striatal neurons. In addition to efforts targeting the causative mutation, promoting mitochondrial health may be a complementary strategy in treating diseases with DNA instability such as HD.

## Teaser

Neueder *et al.* reveal an accumulation of mitochondrial DNA mutations and impairments in mitochondrial quality control in fresh skeletal muscle samples from patients with Huntington’s disease. Accelerated mitochondrial ageing may thus be one of the biological signatures of Huntington’s disease, including in peripheral tissues.

## Introduction

In Huntington’s disease (HD), a CAG repeat expansion mutation in the huntingtin gene (*HTT*) causes a relentlessly progressing mixed movement disorder with dementia and behavioural abnormalities along with a peripheral phenotype involving skeletal muscle.^1^ Mitochondrial dysfunction has been reported in tissues of HD models, including skeletal muscle, and cultures of lymphoblasts and fibroblasts from patients with HD.^2^ Mutant HTT was shown to interact with the large cytosolic GTPase dynamin-related protein 1 (DRP1) that, upon its activation, induces mitochondrial fission, allowing for the removal of mitochondrial network portions that, for instance, contain mutated mitochondrial DNA (mtDNA).^3^ Increased DRP1 activity is associated with increased mitochondrial network fragmentation in human HD fibroblast and lymphoblast cell cultures as well as in HD mouse primary neurons and brain.^4-6^ Consistent with the notion of increased mitochondrial fission in the pathogenesis of HD, the inhibition of DRP1 reverses the phenotype of mitochondrial fragmentation and improves survival in HD mice.^7^ Mutant HTT might in addition impair mitophagy,^8-13^ an evolutionarily conserved quality control system in eukaryotes for selective removal of dysfunctional mitochondria.^14^

Impaired mitochondrial quality control in HD can affect mitochondrial homeostasis, which is essential for mitochondrial function, including ATP production via oxidative phosphorylation and mtDNA stability. In striatum of an HD mouse model overexpressing relative levels of the exon 1 HTT N-terminal fragment (R6/2 mice), levels of mtDNA were reduced,^15^ and in STHdhQ7 and STHdhQ111, mouse striatal cells expressing mutant HTT, more mtDNA lesions were observed.^16^ In leucocytes and fibroblasts from human HTT mutation carriers, mtDNA levels (mtDNA/nuclear DNA ratio) were lower than in controls.^17-19^

Mutations arise at a much higher rate in mtDNA than nuclear DNA.^20^ The number of mtDNA copies harbouring mutations increases with age^21^ and is linked to age-related decline of mitochondrial function in humans.^22,23^ In contrast to dividing cells, post-mitotic cells or tissues such as neurons or skeletal muscle rely on mitochondrial quality control mechanisms to sequester faulty parts of the mitochondrial network by mitochondrial fission, remove them by mitochondrial autophagy (mitophagy) and replace them with new mitochondrial content. Impaired mitochondrial quality control could result in lower mtDNA quality in patients. In diseases such as HD with impaired mitochondrial quality control, a chronic subtle failure of dealing with mutagenic stress could result in higher levels of mutant mtDNA copies than in healthy individuals of similar age.^19^ This is important because accelerated mitochondrial ageing could be a downstream effect of expressing mutant HTT.^24^

Almost all data suggesting mitochondrial involvement in general, and mitochondrial quality control in particular, come from model systems or post-mortem human tissue, in which the events leading to death and post-mortem changes may have affected the data. The examination of fresh human skeletal muscle tissue that relies on mitochondrial health similar to brain can contribute enormously to determining the long-term effects of expressing mutant HTT. In the present work, we addressed this question by examining mtDNA stability and mitochondrial quality control pathways in human skeletal muscle and primary cell lines from *HTT* CAG repeat expansion mutation carriers and matched healthy volunteers. Through systematic analysis of mitochondrial parameters and mtDNA sequences obtained from ultra-deep sequencing, we show an accelerated accumulation of pathogenic mtDNA mutations in mtDNA in somatic tissues in patients with HD and provide mechanistic insight into abnormal mitochondrial network dynamics, illustrating a novel molecular feature underlying HD biology.

## Materials and methods

### Human subjects

Forty-two *HTT* CAG trinucleotide repeat expansion carriers and 24 healthy sex and age-matched controls were recruited at the Departments of Neurology of Ulm University and University College London. All details about demographic and clinical data, as well as the description of the study, have been published alongside the Multiple Tissue Monitoring in Huntington disease (MTM-HD) data paper.^25^

### Cell lines

Primary human fibroblast and myoblast cell lines from the MTM participants were established and maintained as previously described.^26^ HEK293 HTT exon 1 cell generation and maintenance were as previously described.^27^

### Time-resolved fluorescence energy transfer assay for mutant HTT detection

Soluble mutant HTT detection by time-resolved fluorescence energy transfer (TR-FRET) was performed as previously described.^28^ In brief, 5 µl of tissue homogenate were transferred to a low-volume well of a white opaque 384-well microtitre plate (Greiner). Detection buffer (1 µl) containing 1 ng/µl of 2B7-Tb donor antibody and 10 ng/µl of MW1-d2 acceptor in 50 mM NaH_2_PO_4_, 400 mM NaF, 0.1% bovine serum albumin (w/v) and 0.05% Tween (v/v) was added and incubated for 1 h at 300 rpm at room temperature. The TR-FRET signal was analysed with an EnVision Reader (PerkinElmer) using an excitation pulse time delay of 100 ms at 320 nm. The resulting Tb and d2 emission signals were read at 620 and 665 nm, respectively.

### Mitochondrial DNA copy number

DNA was isolated with a Puregene Core Kit A (Qiagen) as instructed by the manufacturer. The mtDNA copy number was determined with quantitative real-time PCR using the CFX384 Touch Real-Time PCR Detection System (Bio-Rad) as previously described.^15^ For the detection of mtDNA, the D-loop region was used. As a reference, *B2M* (beta-2-microglobulin) encoded on the nuclear DNA was used.^29-31^

### Enzyme activity assays

Tissue lysates were prepared as described previously.^15^ Briefly, tissues were homogenized and cleared of cell debris by centrifugation (600*g*, 10 min at 4°C). The supernatant was used for the activity assays. Measurements of the activity of the respiratory chain subunits and of citrate synthase were performed as described previously.^15,32-35^

### Multiple factor analysis

**Figure 1:**
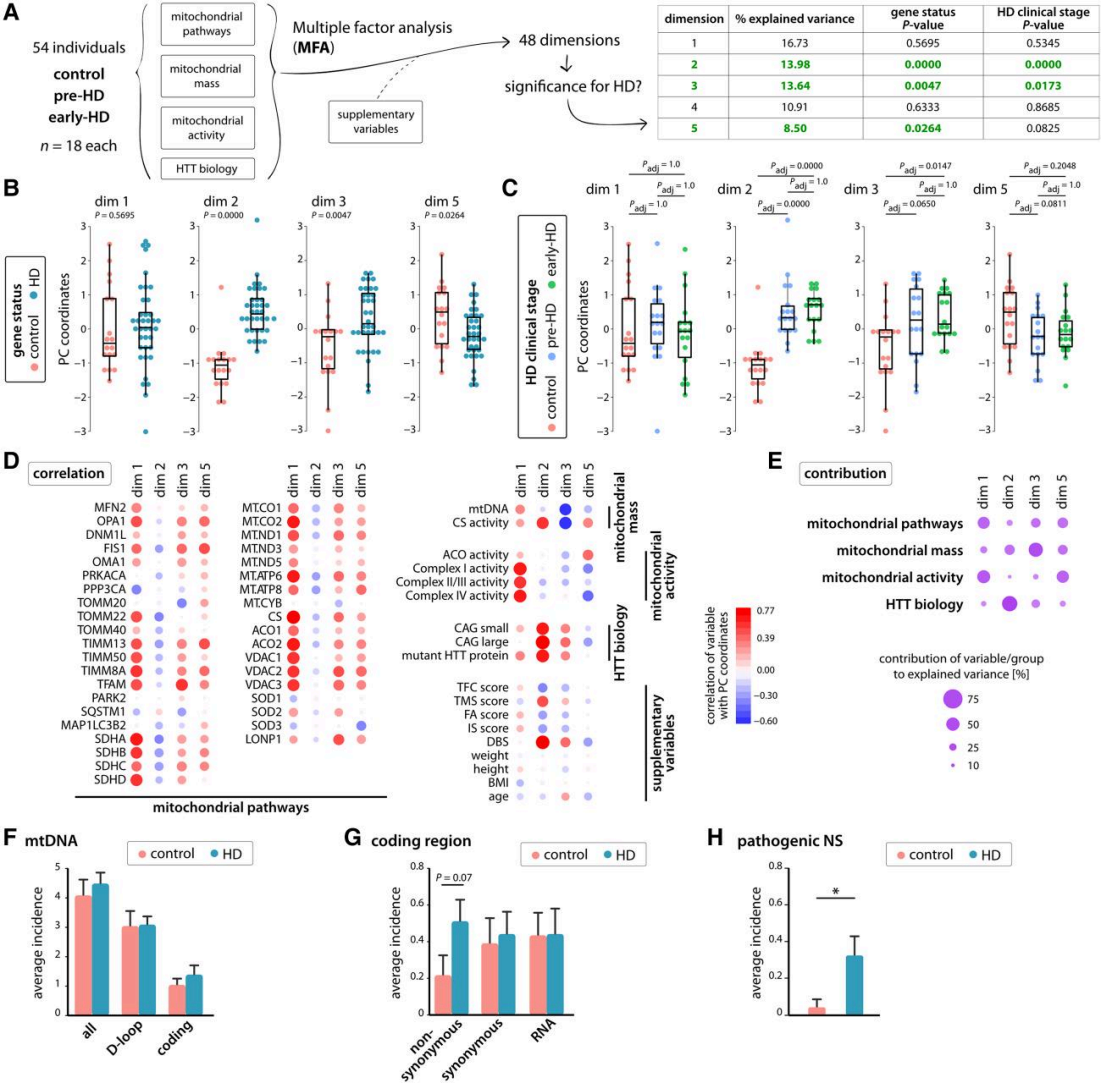
**Multiple factor analysis and mtDNA mutation load in skeletal muscle of Huntington’s disease patients points towards impaired mitochondrial homeostasis**. (**A**) Workflow of the multiple factor analysis (MFA). Using four groups of variables, we defined 48 dimensions in our dataset. Of the first five dimensions, three were significantly correlated with the clinical stage of Huntington’s disease (HD). Principal component (PC) coordinates for each individual are shown for gene status (**B**) and HD clinical stage (**C**) as box plots together with the statistical significance of the group difference (Kruskal–Wallis rank sum test). (**D**) Heat map showing the correlation values for each variable in the four groups against PC dimensions 1, 2, 3 and 5. Red circles are positively correlated and blue circles are negatively correlated. The size and colour intensity of the circles depict the strength of the correlation. (**E**) Plot showing the contribution of each group towards the definition of PC dimensions 1, 2, 3 and 5. The size and colour intensity of the circles depict the amount of contribution towards the respective dimension. (**F**) The average incidence for all mutations, and mutations stratified by their location in mtDNA (D-loop and coding region) detected in each sample. (**G**) The average incidence of mutations in the mtDNA coding region stratified by their functional annotation: non-synonymous mutations and synonymous mutations in the protein-coding region, and mutations in the RNA-coding region. (**H**) The average incidence for non-synonymous (NS) mutations predicted with pathogenicity in each sample. In **F**–**H**, data are presented as mean ± standard error of the mean (SEM). Controls *n* = 23; HD *n* = 43. Statistical analyses were performed using two-tailed homoscedastic Student's *t*-tests. BMI = body mass index; dim = dimension.

Multiple factor analysis (MFA) was conducted using R (v3.5.1) and the indicated modules. We included a total of 54 individuals in the MFA analysis (*n* = 18 each: control, premanifext HD and early stage-HD). We mapped the normalized expression levels of mitochondrial pathway marker proteins from the proteomics analysis, normalized HTT levels, relative mtDNA (to nuclear DNA) and normalized citrate synthase, aconitase, complex I, II/III and IV activities as well as clinical outcome measures. To deal with missing values, multiple imputations using a non-parametric, mixed-type imputation Random Forest algorithm with 100 iterations and 5000 trees were performed.^36^ For MFA analysis, we categorized the variables into four groups as shown in Fig. 1. MFA was computed using FactoMineR^37^ in R (v. 3.5.1). We retained the maximum of 48 dimensions and calculated association with HD status and stage using a Kruskal–Wallis rank sum test (Supplementary Table 1). The results for the quantitative variables can be found in Supplementary Table 2; the results for the supplementary quantitative variables can be found in Supplementary Table 3; and the results for the group analysis can be found in Supplementary Table 4.

### Mitochondrial DNA targeted sequencing and data processing

MtDNA of skeletal muscle samples was sequenced using a targeted approach called STAMP (sequencing by targeted amplification of multiplex probes). Using STAMP, the entire human mtDNA was captured using 46 pairs of probes, each of which contained a unique molecular barcode. The captured products were then amplified with primers targeting common sequences in the 46 pairs of probes. Individual barcodes and sequencing adaptors were added through PCR primers to allow multiplex sequencing. The resulting sequencing libraries were measured with 2 × 250 paired-end reads on a HiSeq 2500 system (Illumina) for samples in the current study. The sequencing reads were processed using a Python pipeline (the STAMP tool kit, available at https://github.com/mtstamp/stamp).^38^ Among the skeletal muscle samples sequenced, we obtained median coverage of unique reads on mtDNA at a depth of about 4200-fold (X) after quality filtering. About half of these unique reads showed sequencing duplicates according to information on the molecular barcode attached to the capture product. Base information from duplicate reads was leveraged to remove sequencing and PCR errors to improve the overall sequencing base quality and reduce error rates. We called mtDNA variants with a variant allele fraction (VAF) ≥ 0.5% in relation to the reference human mtDNA sequence and employed a series of quality control filters on these variants to determine mtDNA mutations. The functional annotation of mtDNA mutations was obtained using the ANNOVAR pipeline.^39^ Pathogenicity predictions were retrieved from the CADD database^40^ (v.1.3) for mtDNA mutations in the protein-coding region. Annotation on reported or confirmed disease association was extracted from the MITOMAP website^41^ and the ClinVar database.^42^ Pathogenic mtDNA mutations in the protein-coding region of mtDNA were defined as having a CADD phred score >20 or disease associations and not to overlap with known mtDNA polymorphisms identified in a healthy general population based on HmtDB.^43^ Statistical analysis of mtDNA mutations was as follows: the incidence of mtDNA mutations was compared between HD and control samples using Student’s *t*-test. For each statistically significant result from the *t*-test, the association was further evaluated using logistic regression or multinomial logistic regression with adjustment for age. The change in mtDNA mutation incidence relative to age was assessed using Poisson regression. All statistical analyses were performed with R (v.3.5.0). Two-tailed *P*-values are reported.

The entire 16.6 kb human mtDNA was captured using STAMP with 46 pairs of probes designed with specific sequences complementary to the heavy or light strand sequences of mtDNA.^38^ The capture reaction was performed on 50 ng total genomic DNA with a diluted extension and ligation (EL) probe pool in 10 µl 1× Ampligase buffer (Epicentre). DNA was first denatured at 95°C for 10 min and then incubated for 20 h after a gradual decrease in temperature at 1°C per min to 55°C to allow hybridization of probes with their target DNA sequences. Six microlitres of gap-filling mix containing 0.1 mM dNTPs, 0.6 M betaine, 0.1 M (NH_4_)_2_SO_4_, 0.5 units of *Tsp* DNA polymerase and 0.5 units of Ampligase in 1×Ampligase buffer were added to the reaction, which was then incubated at 55°C for another 20 h for gap filling. After cooling to 20°C, 1.5 µl of the capture product was used for PCR amplification and individual barcoding in a 50 µl reaction with 1× Phusion HF buffer, 0.5 µM p5i5 and p7i7 indexing primers, 0.2 mM dNTP and 1 unit of Phusion Hot-Start II DNA polymerase (Thermo Fisher Scientific). The thermal conditions were 30 s at 98°C, followed by 25 cycles of 10 s at 98°C, 15 s at 65°C and 15 s at 72°C. The PCR products were then pooled and purified using AMPure XP magnetic beads (Beckman Coulter). The obtained STAMP libraries were sequenced with 2 × 250 paired-end reads on a HiSeq 2500. The Phi-X DNA library was spiked in at 5% to increase the overall complexity of the libraries.

We developed a pipeline in Python to process reads generated from STAMP (available at https://github.com/mtstamp/stamp).^38^ Paired-end reads were first demultiplexed into files for individual samples sequenced in each batch. For each individual sample, paired-end reads were further sorted into clusters of capture products according to the probe arm sequences identified. The arm sequences and molecular barcode were trimmed from the paired-end reads, which were stored as annotations in the read and alignment files. The resulting reads were aligned using the Burrows–Wheeler aligner (bwa mem, v0.7.17)^44^ to the complete human genome, including both nuclear DNA and mtDNA sequences (genome assembly GRCh38, downloaded from ftp://ftp.1000genomes.ebi.ac.uk), and in a second round, to a modified mtDNA sequence, which had the final 120 bp copied to the start to accommodate alignment of D-loop-region reads in STAMP. Paired-end reads that were not aligned to the target region specified by their arm sequences were removed. The remaining reads were locally realigned using freebayes (v1.1.0),^45^ and their base qualities were subsequently recalibrated using samtools (v1.6).^46^

For paired-end reads with the same molecular barcode, the base information at corresponding sites of the alignments was merged using a Bayesian approach to generate a consensus read representing the captured mtDNA product. In brief, the posterior probability of having a nucleotide such as ‘A’ at a certain position in the consensus read can be represented using the following equation:


(1) $$p\left(A|\mathrm{all}\;\mathrm{reads}\right)\;=\;\frac{\prod_{i\;=\;1}^{n}\;p(\mathrm{rea}d_{i}\left|A\right)\times p\left(A\right)}{\sum_{\mathrm{NT}}\;\prod_{i\;=\;1}^{n}\;p(\mathrm{rea}d_{i}\left|\mathrm{NT}\right)\times p\left(\mathrm{NT}\right)}$$

where *p*(NT) is the prior probability and $\overset{n}{\underset{i\;=\;1}{\prod }}\;p(\mathrm{rea}d_{i}\left|\mathrm{NT}\right)$ is the estimated likelihood, under the assumption that all paired-end reads in a read family are independent. To simplify the calculation, we used equal prior probability for all nucleotides. The likelihood of a nucleotide in each read can be approximated using the base quality score as:


(2) $$p(read_{i}|\mathrm{NT})=\;\{\begin{array}{r} 1\text{-}10^{-\frac{\mathrm{BAQ}}{10}},\;\mathrm{NT}="A"\\ \frac{1}{3}\times \;10^{-\frac{\mathrm{BAQ}}{10}},\;\mathrm{NT}\neq "A" \end{array}$$

The nucleotide with the highest posterior probability (*p*_max_) was used to construct the consensus read and assign a quality to this nucleotide using the phred score of its probability as −10log_10_(1−*p*_max_). The quality scores of the consensus read were rounded to the nearest integers and stored in a bam file with ASCII characters from 33 to 126. The same method was also used to merge base information within the overlapping region of the paired-end reads. The sequences of consensus reads were then compared with a collection of known NUMTS (nuclear mitochondrial segment) sequences in the reference genome obtained from a BLASTN search of the 46 mtDNA segments captured with EL probes, as well as their variant sequences harbouring common polymorphisms (minor allele fraction > 1%) identified in the 1000 Genomes project (retrieved from the *commonSNP147* track from the UCSC Genome Browser).^21^ A consensus read was marked as a potential NUMTS if it showed a lower pairwise edit distance to NUMTS sequences than that to the sample’s major mtDNA sequence or if it was constructed from paired-end reads already annotated as NUMTS according to Burrows–Wheeler alignment. Finally, consensus reads were converted to single-end reads, along with their base quality information, and stored in a bam file for each individual sample.

MtDNA variants were determined using consensus reads with a MAPQ (mapping quality score) ≥20 and BAQ (base alignment quality score) ≥30. Consensus reads marked as NUMTS or showing an excess of mismatches (>5 in the coding region and >8 in the D-loop region) compared with the individual’s major mtDNA sequence were also excluded from analysis. To reduce false positive calls of mtDNA mutations, variants were subject to a list of quality filters, including (i) ≥100× depth of coverage with ≥70% of the bases having a BAQ ≥30; (ii) not in low-complexity regions (m.302–m.316, m.512–m.526, m.16184–m.16193); (iii) ≥5 minor alleles detected; (iv) a log likelihood quality score of the variant ≥5; (v) comparable VAFs (Fisher’s exact test *P* ≥ 10^−4^ and fold-change ≤5) computed using consensus reads constructed with or without duplicate paired-end reads (VAF was at least 0.2% among consensus reads constructed with duplicate paired-end reads); and (vi) the detected number of minor alleles significantly larger than the expected number of errors, which was estimated at a rate of 0.02% in STAMP (Exact Poisson test, *P* < 0.01/16569).

Among the samples in the current study, the control samples had slightly higher coverage of consensus reads on mtDNA than the samples from HD patients (average median coverage on mtDNA: 4556× in controls versus 4073× in HD samples; *t*-test, *P* = 0.026). Ninety-seven per cent of mtDNA sites in controls and 96% mtDNA sites in HD samples were covered with over 1000 consensus reads (*t*-test, *P* = 0.12), which were needed to identify a variant of VAF = 0.5%. Moreover, we found that samples from three HD patients had an excess of variants detected at known polymorphic sites in the coding region of mtDNA compared with others (*n* ≥ 11 versus *n* = 0–3 in other samples), which could be caused by low-level contamination of genomic DNA with a different mtDNA sequence. Therefore, we excluded these three samples from the analysis of mtDNA mutations.

### MitoTracker labelling of mitochondria

For myoblast and fibroblast cultures, 5000–10 000 cells were seeded on 0.1% gelatin-coated coverslips in 24-well plates and grown until 60%–80% confluency. For HEK293 cell culture, 10 000 cells were seeded on 0.1% poly-L-lysine coated coverslips in a 24-well plate and grown until 60%–80% confluency. MitoTracker labelling was either with MitoTracker Red (M7512, Invitrogen, 1:9000 of a 0.5 mM stock solution) or Green (M7514, Invitrogen, 1:3000 of a 0.4 mM stock solution) for 45 min at 37°C in Dulbecco’s modified Eagle medium without fetal bovine serum. Cells were subsequently kept in cell culture medium without MitoTracker for 30 min. After fixation with 4% paraformaldehyde, where applicable, desmin staining was performed as described previously.^26^ Coverslips were mounted in Citifluor (R1320, Agar Scientific) containing DAPI (4ʹ,6-diamidino-2-phenylindol dilactate D3571, Life Technologies, 1:1000). Following fluorescence labelling, cells were examined with a Zeiss LSM-700 microscope with 40× oil objectives (for fibroblast and myoblast cultures) and 63× oil objectives (for HEK293 cells).

### Automated mitochondrial network analysis

Automated mitochondrial network analysis was conducted using Fiji.^47^ We used at least six images with multiple cells per cell line for the analysis. For detailed *n* values, see figure legends. The first step of the analysis was channel separation of the MitoTracker and DAPI-stained images. Nuclei were counted with the ‘Analyze Particles’ plugin on the DAPI channel to give the cell number per image. For the MitoTracker stained networks, the following preprocessing filters were run consecutively in this order: (i) Unsharp Mask (radius = 1 mask = 0.60); (ii) Enhance Local Contrast (CLAHE) (block size = 255 histogram = 256 maximum = 2); (iii) Subtract Background (rolling = 10); and (iv) Despeckle. Following this, signals were separated from the background using auto-thresholding with default settings. This generated binary images (signal or no signal/pixel). The sum of the signal normalized to the total image area was defined as the total mitochondrial footprint. In the next steps, the image was binarized and skeletonized. Networks were analysed using the ‘Analyze Skeleton’ plugin.^48^ Subsequent data evaluation was carried out in R (v. 3.5.2). Data from the analysis of the skeletonized networks were imported, combined and screened for abnormal images using a Grubbs test (part of the ‘outliers’ package).^49^ Subsequently, data for images were aggregated per cell line and used for statistical evaluation of differences between genotypes.

### Western blot analysis

Cells were harvested in lysis buffer [5% (w/v) sodium dodecyl sulphate, 200 mM Tris (pH 6.8), 1 mM EDTA (pH 8), 215 mM β-mercaptoethanol, 8 M urea supplemented with cOmplete™ Protease Inhibitors (Sigma Aldrich, 11697498001)]. The lysate was sonicated until viscosity was visibly reduced. Protein concentration was measured on a Nanodrop 1000 system (Thermo Fisher). Protein concentrations were adjusted to approximately 2 mg/ml, and 20 µg of protein were loaded on pre-cast gels (Criterion XT Bis-Tris, Bio-Rad, 3450113 and 3450125). Proteins were transferred onto a nitrocellulose membrane and incubated with primary antibodies in Tris-buffered saline with Tween 20 [TBS-T; 20 mM Tris (pH 7.4), 150 mM NaCl, 0.1% (v/v) Tween 20] overnight at 4°C. Antibodies were as follows: anti-TUBB (Sigma-Aldrich T7816, 1:20000); anti-SDHA (Abcam ab14715, 1:10000); anti-MFN1 (Abcam ab57602, 1:500); anti-MFN2 (Abcam ab56889, 1:500); anti-OPA1 (BD Biosciences 612606, 1:500); anti-PINK1 (Novus Biologicals BC100-494, 1:750); anti-DNM1L (Cell Signalling 5391S, 1:750); anti-DNM1L *P*-S616 (Cell Signalling 3455S, 1:750); anti-SQSTM1 (Cell Signalling 88588S, 1:1000); and anti-CASP3 (Cell Signalling 9662, 1:2000). After three washes with TBS-T, membranes were incubated with secondary antibodies [rabbit anti-goat IgG heavy chain (H) + light chain (L) (Thermo Fisher, R21459) or goat anti-mouse IgG (H + L) (Bio-Rad, 172–1011)] for 45 min at room temperature. After three washes with TBS-T, membranes were developed with Luminata Forte Western HRP Substrate (Merck Millipore, WBLUF0500) and signals were acquired on a LAS4000 system (GE Healthcare). Signal quantification was performed using Li-Cor Image Studio Software v5.2.

### Quantification and statistical analysis

All data were evaluated blinded to the samples’ genotype. Statistical analysis was performed using SPSS (v.25, IBM) and R (v. 3.5.1 and 3.5.2). Data were examined for outliers using a Grubbs test in R (part of the ‘outliers’ package).^49^ Statistical analysis of two groups was carried out using an unpaired Students *t*-test or ANOVA for more than two groups followed by Tukey’s *post hoc* test. Detailed descriptions including *n* values can be found in the figure legends.

## Results

### Impaired mitochondrial DNA maintenance in human HD skeletal muscle

Skeletal muscle tissue was obtained by open biopsy of the vastus lateralis muscle from participants carrying the *HTT* CAG repeat expansion mutation and age- and sex-matched healthy volunteers. These samples were collected under the MTM-HD study^25^ (see the ‘Materials and methods’ section). To examine how clinical, demographic, HD biology and mitochondrial data related to each other, we used MFA. MFA is similar to a principal component analysis (PCA) that allows relationships in complex data to be analysed in multiple dimensions. Each dimension explains the variance in the residuals of the previous dimension. In contrast to PCA, MFA uses grouped variables that define the PCA and gives the same overall weight to the variables for each group. This balances the influence of each group in the overall analysis. Hence, MFA offers the advantage of probing the relationships between the groups of variables and their contribution to the variance in the dataset, rather than just examining the relationship between individual variables as in conventional PCA. Moreover, the internal structure of variables/data in each group is preserved.

In our MFA, we examined the relationship between four groups: mitochondrial pathways (variables: protein expression data by proteomics of 39 proteins); mitochondrial mass indicators (variables: mtDNA copy number and citrate synthase activity); mitochondrial activity (variables: mitochondrial respiratory chain complex activities); and HD biology (huntingtin CAG repeat lengths, mutant huntingtin protein levels) (Fig. 1A and Supplementary Table 6). Demographics (variables: age, weight, height) and clinical measures [Unified Huntington Disease Rating Scale variables: total functional capacity, total motor score and function analysis (independence scale)] were used as supplementary variables. Supplementary variables do not contribute to the principal components, also called dimensions, of the MFA but can be used to calculate correlations (Fig. 1A).

Of the 48 total dimensions, the first five explained 64% of the total variance (Fig. 1A and Supplementary Table 1). Dimension 1 (16.7% variance explained), common to HD participants and healthy volunteers, reflected differences in mitochondrial biology in the general population, independent of HD (Fig. 1B and C). The main discriminator was an increase in mitochondrial proteins, concomitant with increased mitochondrial respiratory chain complex activity and, to a lower extent, increased mitochondrial mass (Fig. 1D and E and Supplementary Tables 2 and 3). Three of the next four components significantly differed between HD and controls, as well as between premanifest HD, manifest HD and controls (Fig. 1B and C). However, we did not detect significant differences between premanifest and early-stage HD groups (Fig. 1C). Dimension 2 explained 14% of data variability, which was driven mainly by key HD parameters such as CAG repeat length, mutant HTT levels and disease burden (CAG-35.5 × age)^50^ as well as citrate synthase activity as a measure of mitochondrial mass. With higher CAG repeat length and higher mutant HTT levels, mitochondrial mass was also higher (Fig. 1D and E). Dimension 3 explained nearly 14% of data variability and distinguished HD from controls. Protein expression levels of TFAM, LonP, VDAC2 and VDAC3 were positively correlated, while mtDNA copy number and citrate synthase activity were negatively correlated. Thus, with lower mtDNA copy number and mitochondrial mass, TFAM, LonP and VDAC2/3 protein levels were higher. HD cases had higher principal component scores, suggesting a shift towards lower mtDNA copy number and mass, and higher TFAM, LonP and VDAC2/3 protein levels than healthy volunteers. In HD, this points towards possible impairments in mtDNA maintenance, with a consequent increase in mitochondrial biogenesis (TFAM) and amounts of VDAC2/3 and LonP as essential regulators of mtDNA copy number that might contribute to lower mtDNA levels and retention of deleterious mtDNA mutations.^51,52^ This was supported by Dimension 5, which distinguished cases and controls, explaining 8.5% of the variability. In this dimension TIMM13, FIS1, OPA1, VDAC and aconitase activities correlated positively with HD status, while complex I and IV activities correlated negatively (Fig. 1D and E and Supplementary Table 4). Principal component scores were higher in controls than HD, suggesting patients had less complex IV activity relative to levels of important inner and outer mitochondrial membrane and fission regulator proteins and TCA cycle activity.

Taken together, the dimensions identified by the layered analysis using MFA pointed towards differences between HD and controls within the mitochondrial network. The dimension explaining most of the variability indicated that the largest proportion of the mitochondrial network was similar in controls and patients, except for a larger mitochondrial mass in patients. However, the deeper layers of the MFA analysis reflected relationships in other sections of the mitochondrial network in patients, in which mtDNA was depleted and complex IV activity lower than in controls, while levels of important components of mitochondrial quality control were, potentially in response, higher than in controls.

### Predicted pathogenic non-synonymous mtDNA mutations are more frequent in HD skeletal muscle

The steady-state data in human HD skeletal muscle indicate mtDNA homeostasis is compromised. Therefore, we next asked whether life-long expression of mutant HTT could lead to an accumulation of mtDNA mutations in HD participants. We analysed mtDNA mutations in 66 skeletal muscle samples using ultradeep mtDNA sequencing.^19,38^

The average number of mtDNA mutations in muscle was 4.1 in control individuals and 4.5 in HD patients, which was not significantly different between the 43 HD patients and 23 controls (Supplementary Table 5). The majority (71%) of mutations were detected in the D-loop region of mtDNA, with the most abundant ones at m.72T>C, m.189A>G and m.408T>A (Fig. 1F). These mutational hotspots were in accordance with those identified in a previous study on mtDNA of skeletal muscle samples.^53^ We found that HD patients had slightly increased numbers of mutations in both the D-loop region and coding region of mtDNA than controls, but the differences were not statistically significant (Fig. 1F). mtDNA mutations in the coding region that alter protein sequences of mtDNA-encoded genes (non-synonymous mutations) showed a non-significant increase of >1-fold in HD patients compared with those in controls (*t*-test, *P* = 0.07), while the incidence of synonymous mutations and those in the RNA-coding regions was similar in these two groups (*P* ≥ 0.78; Fig. 1G). Next, we assessed the pathogenicity of mtDNA mutations in skeletal muscle samples of HD patients. We found that all non-synonymous mutations in early-stage HD and around half of non-synonymous mutations in premanifest HD patients were predicted to be pathogenic. In contrast, only one of the five non-synonymous mutations identified in the controls was likely pathogenic. That mutation had a low VAF of about 0.6% in skeletal muscle. As a result, HD patients carried six times more predicted pathogenic non-synonymous mutations in skeletal muscle than controls (*P* = 0.015; Fig. 1H).

Next, we compared mtDNA mutations identified in the current study with those from the study by Li *et al*.,^53^ in which mtDNA from 150 skeletal muscle samples from the general population was sequenced at an average coverage of 3500× and mutations were called at a VAF ≥ 0.5%.^53^ Because the study by Li *et al*.^53^ included participants with a wide age range, from 3 days after birth to 96 years, comparing them to a study of participants with a narrower age distribution may yield spurious results. We therefore focused on samples from 57 participants aged between 30 and 60 years in the study by Li *et al*.^73^ for further analysis. This age range was similar to the age range of participants in the current study. The average mtDNA mutation incidence in the D-loop region, as well as in the coding region, did not differ significantly between samples in the study by Li *et al*.^53^ and control samples in the current study (Table 1). The increase in non-synonymous mutations predicted to be pathogenic in HD patients remained significant when compared with controls that consisted of samples from the study by Li *et al*.^53^ and control samples from the current study (*t*-test: *P* = 0.015; logistic regression: *P* = 0.031; Table 1). A further multinomial logistic regression analysis indicated that the incidence of pathogenic non-synonymous mutations was significantly associated with early-stage HD [odds ratio (OR) = 3.2, *P* = 0.018] but not with premanifest HD (OR = 2.1, *P* = 0.16). In addition, we observed an age-dependent significant increase of mutations in the D-loop region in both controls and HD patients in accordance with data from the study by Li *et al*.^53^ (Poisson regression: *P* ≤ 0.018; Table 2). Moreover, an age-dependent decrease of mutations in the coding region was found to be significant only in HD patients (Poisson regression: *P* = 0.016; Table 2).

**Table 1:** **Comparisons with skeletal muscle mitochondrial DNA mutations reported by Li *et al***.^53^

	Controls		Huntington’s disease patients		Controls versus Huntington’s disease
Variables	Li *et al*.^a^	Control	Premanifest	Early-stage	*P*-value^b^
	(*n* = 57)	(*n* = 23)	(*n* = 22)	(*n* = 21)	
Age	48 (32–59)	40 (31–55)	41 (31–56)	46 (35–58)	**0**.**041**
All mitochondrial DNA mutations	3.18 (0–8)	4.09 (1–12)	4.05 (1–7)	4.95 (1–12)	0.54
Mutations in the D-loop region	2.54 (0–7)	3.04 (0–12)	2.91 (0–5)	3.29 (0–7)	0.93
Mutations in the coding region	0.63 (0–3)	1.04 (0–4)	1.14 (0–3)	1.67 (0–12)	0.35
Pathogenic non-synonymous mutations	0.11 (0–2)	0.04 (0–1)	0.27 (0–2)	0.38 (0–3)	**0**.**015**

Data are presented as mean (range). *P*-values in bold indicate statistically significant differences.

^a^Skeletal muscle samples from 57 participants aged between 30 and 60 years in the study by Li *et al*.^53^

^b^ *P*-values for Student's *t*-tests on all control samples (*n* = 80) and samples from individuals with Huntington’s disease (*n* = 43).

**Table 2:** Age-dependent changes in mitochondrial DNA mutation incidence in skeletal muscle samples

	Li *et al*.^a^ (*n* = 57)		Controls (*n*= 23)		HD (*n* = 47)	
Variables	Beta (SE)	*P*-value	Beta (SE)	*P*-value	Beta (SE)	*P*-value
All mitochondrial DNA mutations	0.027 (0.011)	0.019	0.043 (0.017)	0.0087	0.01 (0.011)	0.36
Mutations in the D-loop region	0.034 (0.013)	0.0093	0.057 (0.019)	0.0024	0.032 (0.014)	**0**.**018**
Mutations in the coding region	0.0011 (0.024)	0.96	−0.00037 (0.035)	0.99	−0.050 (0.021)	**0**.**016**
Pathogenic non-synonymous mutations	−0.041 (0.055)	0.45	−0.009 (0.17)	0.96	−0.048 (0.043)	0.26

Beta, standard error (SE) and *P*-values are from a Poisson regression of individual age on the number of mitochondrial DNA mutations. HD = Participants with Huntington’s disease. *P*-values in bold indicate statistically significant differences.

^a^Skeletal muscle samples from 57 participants aged between 30 and 60 years in the study by Li *et al*.^53^

### N-terminal HTT fragment expression leads to a pro-fission mitochondrial network phenotype

**Figure 2:**
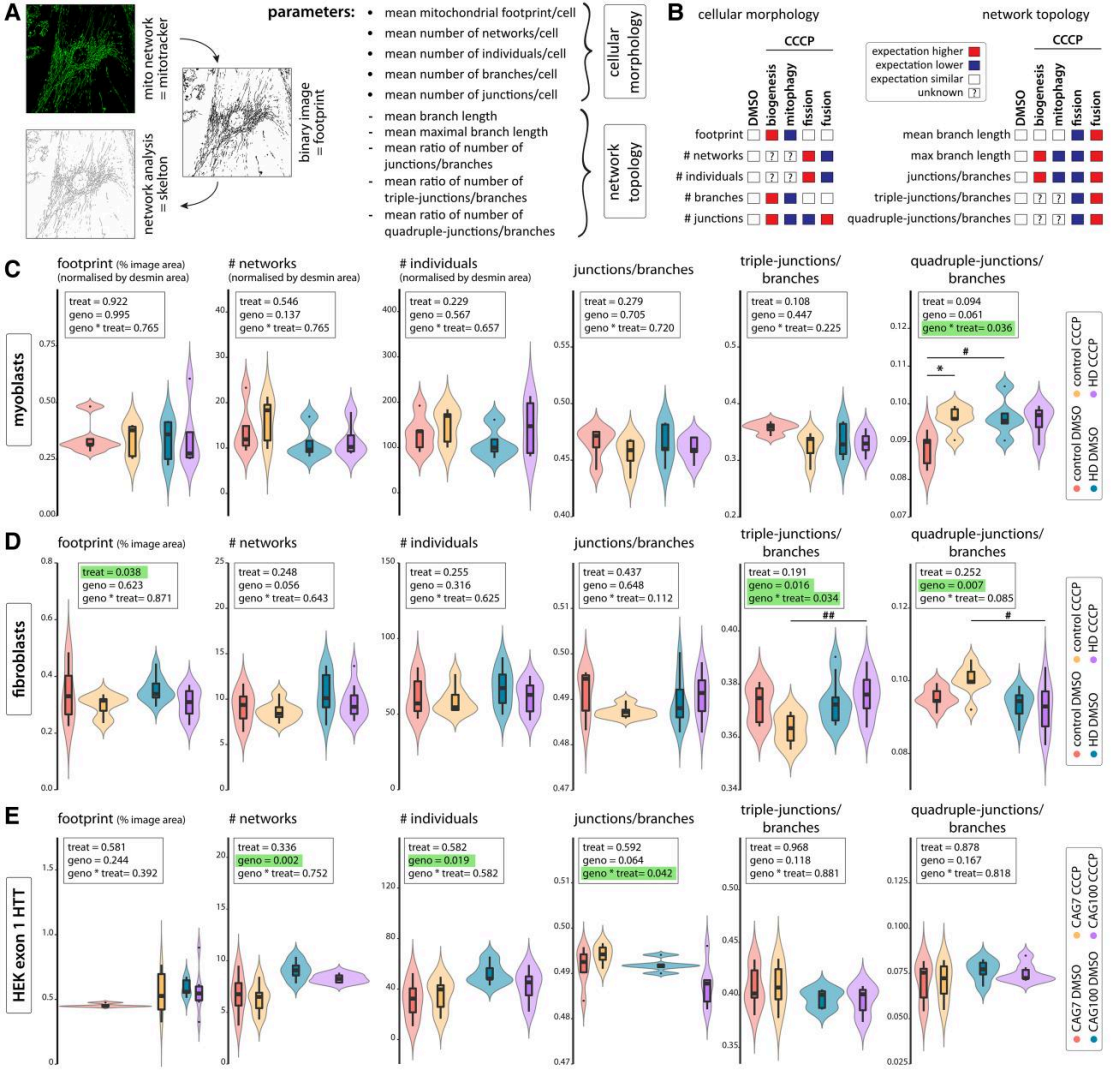
**Mitochondrial network analysis in cellular models of Huntington’s disease (HD) shows changes in higher-order network topology**. (**A**) Schematic showing the analysis of mitochondrial networks and the analysed parameters. For a more detailed description of the methodology, see the ‘Materials and methods’ section and Supplementary Fig. 1. (**B**) Expected direction of changes in the measured parameters upon induction of mitochondrial stress through CCCP treatment. Network analysis results from human myoblasts (**C**), human fibroblasts (**D**) and a HEK293 cell line expressing exon 1 HTT with 7 (CAG_7_) or 100 (CAG_100_) CAG repeats (HEK exon 1 HTT) (**E**). Cells were either treated with DMSO, or mitochondrial stress was induced for 24 h by treatment with 10 µM CCCP. For myoblasts (**C**), only desmin-positive cells were analysed and data was normalized to the desmin area (cell area). Data are presented as violin plots with inlaid box plots. Coloured areas on violin plots show the full distribution of all measured data. Statistical analyses were performed using two-way ANOVA with Tukey’s *post hoc* test. The main effects of treatment (treat, 10 µM CCCP versus DMSO) and genotype (geno; HD versus control), including the interaction of treatment and genotype (geno × treat), are shown. Treatment: *P < 0.05; Genotype: ^#^*P* < 0.05, ^##^*P* < 0.01. Myoblasts: control *n* = 5; HD *n* = 5. Fibroblasts: control *n* = 7; HD *n* = 11. HEK exon 1: CAG_7_/CAG_100_  *n* = 6/6 of independent experiments. The remaining parameters are shown in Supplementary Figs 2 (fibroblasts), 3 (myoblasts) and 4 (HEK exon 1). CCCP = carbonyl cyanide m-chlorophenyl hydrazine; DMSO = dimethylsulphoxide.

The skeletal muscle data indicated a compromise in maintaining mtDNA homeostasis associated with an increase in mtDNA mutations predicted to be pathogenic. Previously, mitochondrial fragmentation had been observed in HD fibroblast and lymphoblast cell cultures.^5,6^ We therefore used primary fibroblast and myoblast cell lines to examine mitochondrial network morphology. Using automated mitochondrial network analysis (Fig. 2A and Supplementary Fig. 1), we generated various readouts to probe the structure and topology of the mitochondrial networks in these samples. These readouts were expected to deviate from the unstressed condition when the mitochondrial membrane potential dissipated following carbonyl cyanide m-chlorophenyl hydrazine (CCCP) treatment (Fig. 2B).

Our analysis showed that mitochondrial morphology in patient-derived myoblast (Fig. 2C and Supplementary Fig. 3) or fibroblast (Fig. 2D and Supplementary Fig. 2) cultures was similar to controls, indicating that the expression of full-length mutant HTT had no effect on the mitochondrial network (Fig. 2C and D). Following exposure to 10 µM CCCP, in control cells [myoblasts (Fig. 2C) or fibroblasts (Fig. 2D)], we observed a loss of mitochondrial footprint, a reduction in triple junctions/branches and an increase in the ratio of quadruple junctions/branches. These network changes are indicative of mitochondrial damage. Higher doses of CCCP (20 and 100 µM) led to massive network fission and cell death in the control fibroblast lines (Supplementary Fig. 2). Therefore, 10 µM CCCP were used for subsequent experiments because this concentration induced mitochondrial stress while not causing cell death.

In contrast to the control cells, where we saw a shift towards higher order network topology with 10 µM CCCP, such a response was not observed in the patient cell lines (Fig. 2C and D, 10 µM CCCP). However, in the myoblast cell lines, already under unstressed conditions, higher order network topology was significantly increased in comparison with the control cells, suggesting that the patient cells exhibited stress that resembled that induced by CCCP exposure (Fig. 2C, quadruple junctions).

We next used human embryonic kidney (HEK) cell lines expressing an exon 1 HTT fragment with 7 (CAG_7_) or 100 (CAG_100_) CAG repeats to investigate the effects of N-terminal HTT fragment expression. These cells were generated using the Flp-IN™ System, which allowed the stable expression of transgenes through integration into the same genomic locus. The HEK cell lines expressed mouse *Htt* exon 1 under control of the endogenous *Htt* promoter.^27^ A highly similar construct was used to generate the R6/2 mouse model, which is the murine model of HD with the most pronounced and rapidly progressing phenotype.^54^ We hypothesized that the extreme pathogenicity of exon 1 HTT represented the maximum possible deviation from normal physiology and thus would exhibit the most pronounced phenotypes. Indeed, at steady-state level, we observed a more fragmented state of mitochondrial networks in the mutant cells compared with the control cells (Fig. 2E and Supplementary Fig. 4), while the stress due to 10 µM CCCP treatment induced no further changes (Fig. 2E and Supplementary material).

### Mitophagy occurs independently of mitofusin ubiquitination in exon 1 HTT fragment-expressing cells

**Figure 3:**
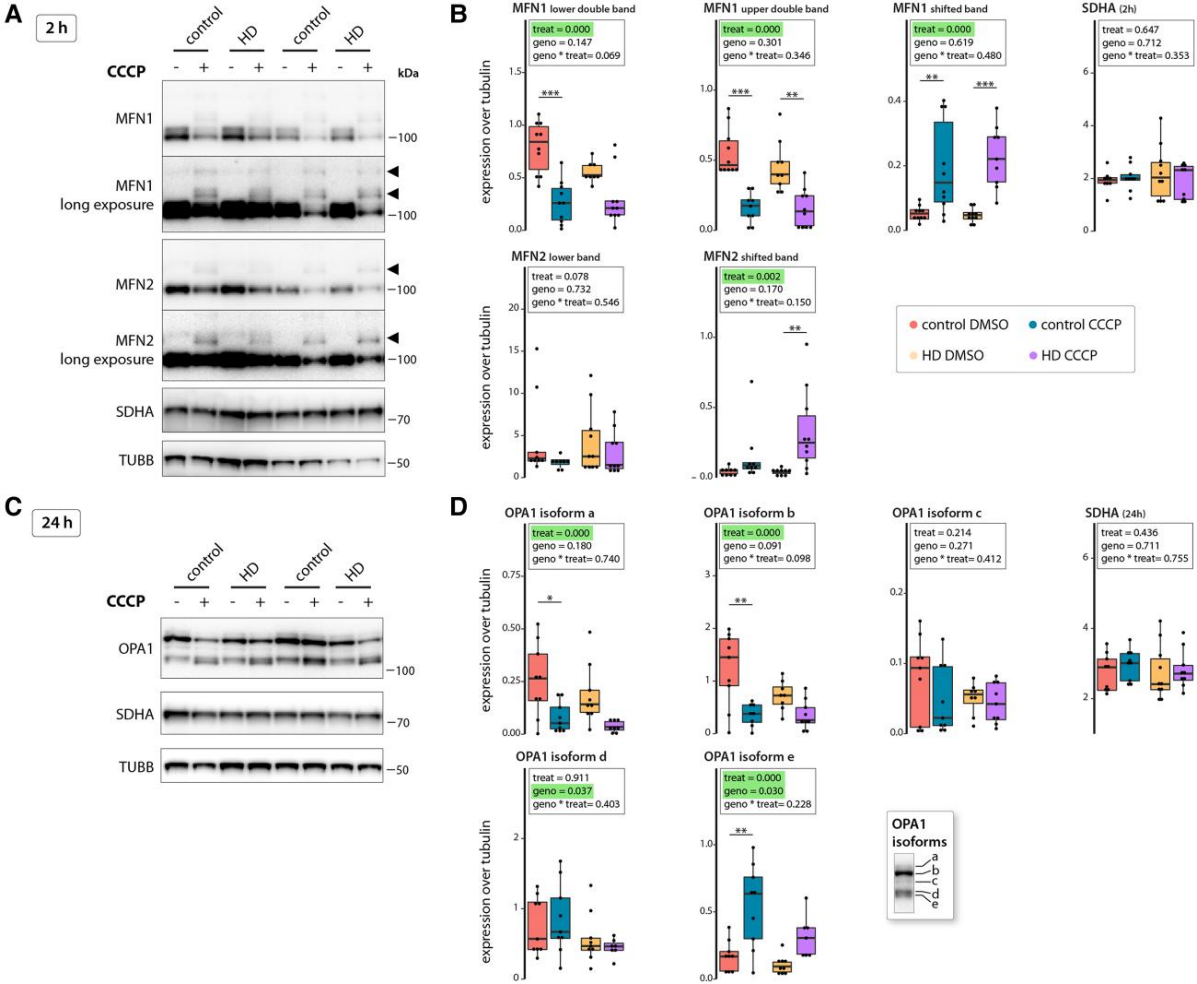
**The response to mitochondrial stress is diminished in Huntington’s disease fibroblasts.** Protein expression analysis of human fibroblasts after induction of mitochondrial stress by treatment with 10 µM CCCP for 2 h (**A** and **B**) or 24 h (**C** and **D**). (**A**) Western blot images for mitofusin 1 (MFN1), mitofusin 2 (MFN2), succinate dehydrogenase complex subunit A (SDHA), and tubulin (TUBB). Filled arrowheads indicate super-shifted proteins due to post-translational modifications. (**B**) Quantification of protein expression levels shown in **A**, normalized to tubulin. (**C**) Western blot images for OPA1 mitochondrial dynamin-like GTPase (OPA1) isoforms (see also *inset* in **D**), SDHA and tubulin. (**D**) Quantification of protein expression levels shown in **C**, normalized to tubulin. (**B** and **D**) Data are presented in box plots. *n* = 10 for all groups. Statistical analyses were performed using two-way ANOVA with Tukey’s *post hoc* test. The main effects of treatment (treat, 10 µM CCCP versus DMSO) and genotype (geno, HD versus control) including the interaction of treatment and genotype (geno × treat) are shown. Treatment: *P < 0.05, **P < 0.01, ***P < 0.001. CCCP = carbonyl cyanide m-chlorophenyl hydrazine; HD = Huntington’s disease.

We next assessed the signatures of important proteins associated with mitochondrial quality control. Protein levels of the OPA1 isoforms d and e were reduced in mutant fibroblasts under basal conditions compared with control cells (Fig. 3C and D). After 2-h of exposure to 10 µM CCCP, in both mutant and control cells, MFN1 and 2 levels were decreased and a larger band appeared, indicative of ubiquitination (Fig. 3A and B). Immunoprecipitation experiments with anti-MFN1 and anti-MFN2 antibodies, followed by western blot analyses with anti-ubiquitin antibodies, have demonstrated that the slower migrating bands recognised by anti-MFN1/2 antibodies in CCCP-treated cells represent ubiquitinated MFN1/2.^55^

After 24 h of exposure to CCCP, levels of the long OPA1 isoforms were decreased (Fig. 3C and D, isoforms a and b) and the levels of the short isoform e were increased. This indicated that CCCP shifted the balance towards mitochondrial fission-promoting proteins but did not induce mitophagy, given that SDHA levels remained similar after short (Fig. 3A and B) or long (Fig. 3C and D) CCCP exposure.

**Figure 4:**
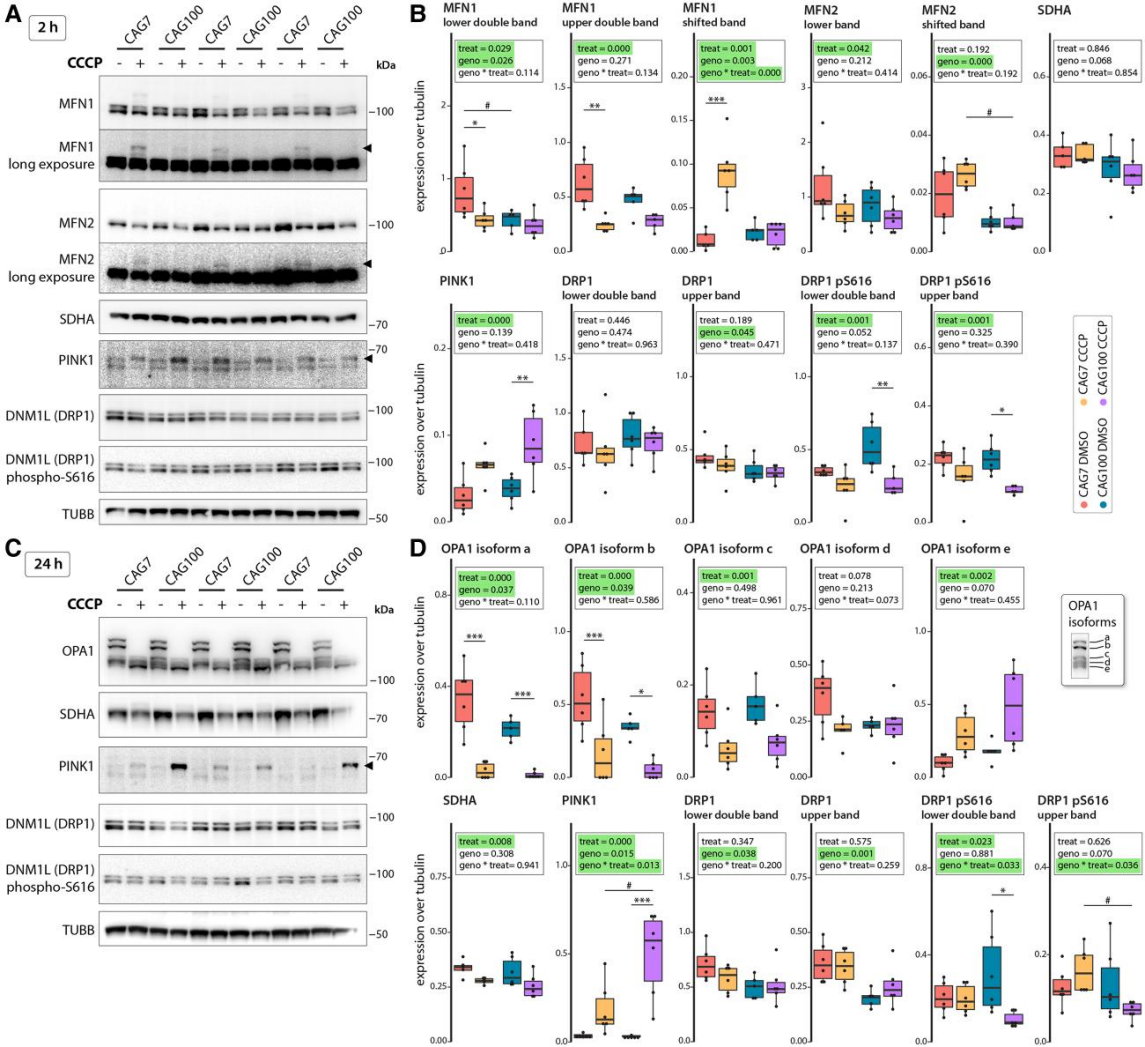
**Expression of exon 1 HTT further exacerbates the diminished response to mitochondrial stress.** Protein expression analysis of a HEK293 cell line expressing exon 1 HTT with 7 (CAG_7_) or 100 (CAG_100_) CAG repeats after induction of mitochondrial stress by treatment with 10 µM CCCP for 2 h (**A** and **B**) or 24 h (**C** and **D**). (**A**) Western blot images for mitofusin 1 (MFN1), mitofusin 2 (MFN2), succinate dehydrogenase complex, subunit A (SDHA), PTEN induced kinase 1 (PINK1), dynamin-related protein 1 like (DNM1L/DRP1) and tubulin (TUBB). Filled arrowheads indicate super-shifted proteins due to post-translational modifications. (**B**) Quantification of protein expression levels shown in **A**, normalized to tubulin. (**C**) Western blot images for OPA1 mitochondrial dynamin-like GTPase (OPA1) isoforms (see also *inset* in **D**), SDHA, PINK1, DNM1L and TUBB. Filled arrowhead indicates super-shifted proteins due to post-translational modifications. (**D**) Quantification of protein expression levels shown in **C**, normalized to tubulin. (**B** and **D**) Data are presented in box plots. *n* = 6 independent experiments for all groups. Statistical analyses were performed using two-way ANOVA with Tukey’s *post hoc* test. The main effects of treatment (treat, 10 µM CCCP versus DMSO) and genotype (geno, CAG_100_ versus CAG_7_), including the interaction between treatment and genotype (geno × treat), are shown. Treatment: *P < 0.05, **P < 0.01, ***P < 0.001; Genotype: ^#^*P* < 0.05. CCCP = carbonyl cyanide m-chlorophenyl hydrazine.

In the exon 1 HTT fragment-expressing HEK cells, mitofusin 1 levels (lower double band) were higher at baseline in control (CAG_7_) cells than mutant cells (Fig. 4A and B). Mitofusin 1 levels were reduced to the levels of CAG_100_ by 2 h of CCCP exposure. One explanation for this could be ongoing fission and mitophagy in the mutant cells. However, baseline levels of mitofusin 2, SDHA, caspase 3, p62, PINK1 and DRP1, and the form of DRP1 phosphorylated at serine 616 indicative of mitochondrial fission activation (*P*-DRP 1), were similar (Fig. 4 and Supplementary Fig. 6). In control cells, 2 h of CCCP treatment resulted in reduced mitofusin 1 and 2 levels (lower and upper double band), with a higher molecular weight band appearing, suggesting ubiquitination (Fig. 4A). In contrast, in mutant cells under steady state levels, there was very little ubiquitinated mitofusin present (Fig. 4A and B). Nonetheless, PINK1 levels increased with CCCP exposure in controls and HD cells, while total DRP1 levels were similar with CCCP exposure and *P*-DRP1 levels decreased in controls and HD cells, indicative of the induction of fission and mitophagy. Unchanged levels of SDHA, p62 and pro-caspase 3, and the absence of (cleaved) caspase 3, suggested that this did not result in a major loss of mitochondrial mass or apoptosis upon CCCP exposure (Fig. 4A and B and Supplementary Fig. 6).

At 24 h of CCCP-induced stress, PINK1 was more stable, with significantly higher levels in the CAG_100_ cells than controls (Fig. 4C and D). In contrast to the 2-h time point, *P*-DRP1 levels were similar in controls and CAG_100_ cells (Fig. 4D). For the OPA1 isoform pattern, following CCCP treatment, we observed a similar phenomenon as in the fibroblast lines: a shift from the longer isoforms towards the shorter isoforms (Fig. 4C and D). Notably, under non-stressed conditions, the CAG_100_ cells expressed lower levels of the longer OPA1 isoforms a and b (Fig. 4D). SDHA levels were lower in both controls and HD upon CCCP treatment (Fig. 4D), while there was no effect on caspase 3 or p62 levels, suggesting that the concentrations of CCCP we used induced mitophagy but not apoptosis or general autophagy (Supplementary Fig. 6).

## Discussion

Recent findings from genome-wide association studies have indicated that DNA repair promotes somatic nuclear *HTT* CAG repeat expansion in the most vulnerable cells in HD (somatic instability).^56^ The data presented in this paper indicate that the topic of DNA instability in HD extends to the second mammalian genome, the mtDNA. MtDNA is crucial to maintaining mitochondrial homeostasis. Therefore, the amount of mtDNA and its genomic integrity are vital for preserving human health.^57^ The depletion of mtDNA can cause severe childhood-onset disorders involving the brain, liver and skeletal muscle,^58^ and mtDNA mutations cause several different diseases, often with adult onset.^57^ In the most comprehensive analyses to date of fresh tissue from human HD, a greater proportion of skeletal muscle mitochondria showed a loss of mtDNA than seen in controls, similar to that previously reported in leucocytes^17^ and the striatum of the N-terminal HTT fragment R6/2 mouse model.^15^ While a peripheral phenotype exists in HD,^1^ skeletal muscle is not as vulnerable as brain tissue, in particular the striatum. However, similar to the brain, skeletal muscle is post-mitotic, expresses mutant HTT from birth and depends heavily on mitochondrial respiration and thus healthy mtDNA. We show that HD skeletal muscle with a loss of mtDNA had higher TFAM and LonP levels in its mitochondria, possibly in an effort to increase mtDNA replication and stability and maintain TFAM:mtDNA ratios.^52^ However, LonP slows mtDNA replication in normal mtDNA but is less efficient when mtDNA harbours mutations, at least in worms.^51^ Higher LonP levels in HD may therefore contribute to lower levels of healthy mtDNA, while maintaining levels of deleterious mtDNA.

Compensatory upregulation of mitochondrial mass to maintain a constant ATP supply increases with a higher biological HD load. However, in contrast to controls, in HD patients with increased mitochondrial mass, this did not translate to more complex IV activity, suggesting efforts to promote mitochondrial biogenesis come with lower efficiency of the mitochondrial respiratory chain in HD.

**Figure 5:**
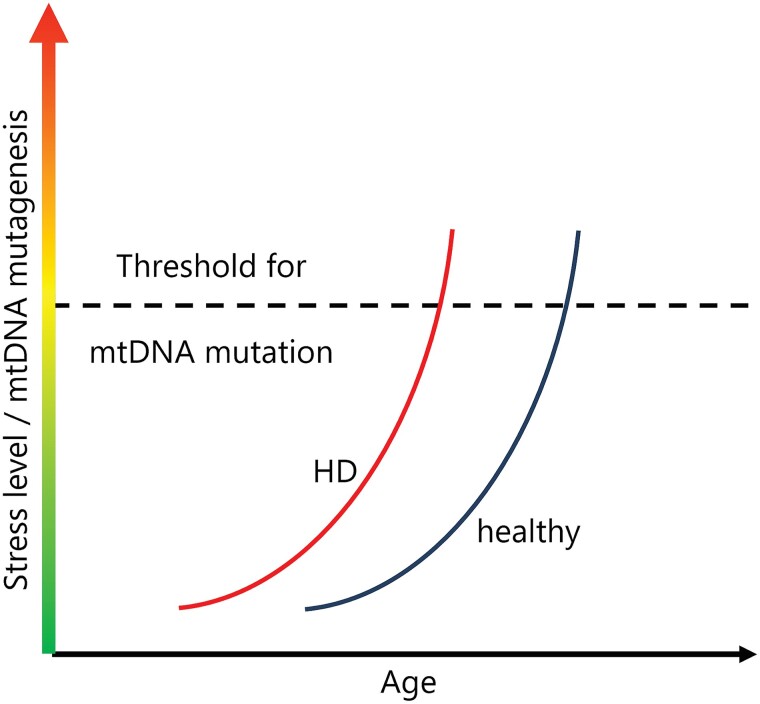
**Schematic of mitochondrial DNA homeostasis in Huntington’s disease**. The expression of mutant HTT affects mitochondrial network dynamics and renders mitochondrial DNA (mtDNA) more vulnerable to stress that induces mtDNA mutations. In Huntington’s disease (HD), pathogenic mtDNA mutations therefore accumulate at a younger age than in healthy individuals.

We show as a key novel finding that predicted pathogenic non-synonymous mtDNA mutations are more frequent in HD skeletal muscle than in age-matched healthy controls. This agrees well with what we found in lymphoblasts from HD patients,^19^ indicating that mtDNA becomes unstable over an HD patient's lifetime. Ageing may drive mutation accumulation in the D-loop region of mtDNA in human skeletal muscle, possibly leading to an adaptive change in mtDNA secondary structure and regulation of mtDNA replication.^53^ The lack of association of mutations in the D-loop region with HD indicated that mitochondrial gene mutations in HD samples arise largely independently of the normal ageing process. The increased mtDNA pathogenicity observed in HD samples implied a deterioration of mtDNA stability in the skeletal muscle of HD patients. This points to lifelong impairments of mitochondrial quality control and maintenance (Fig. 5).

Our investigation of potential mechanisms indicated that the expression of mutHTT promotes mitochondrial fission depending on the HTT CAG repeat size. In primary myoblast or fibroblast lines from patients with full-length expression of HTT and CAG repeat lengths between 40 and 50, there was no evidence of network fragmentation, DRP1 activation or OPA1 fragmentation. In cells with 100 CAG repeats that over-express the most pathogenic N-terminal fragment of mutant HTT, however, mitochondrial networks were fragmented, similar to what has been observed, depending on the HTT length, in HD fibroblasts, neurons and lymphoblast cell cultures^4-6,59^ and in mouse primary neurons.^60^ The HTT mutation itself may promote mitochondrial fission when the amount of N-terminal fragment expression is high, e.g. through alternative splicing, which increases with longer CAG repeat expansions.^27,61-65^ When damaged mitochondria are targeted for mitophagy following fission, PINK1 imported from the cytosol stabilizes on the mitochondrial surface and in turn activates the E3 ubiquitin ligase parkin to promote ubiquitination of various mitochondrial outer membrane substrates.^14,66^ In primary human cell lines expressing full-length mutant HTT, fission-inducing mitochondrial stress was followed by normal ubiquitination of mitochondrial outer membrane proteins with PINK1 stabilization to mediate mitophagy. In contrast, with expression of large amounts of N-terminal mutant HTT fragments following mitochondrial stress, ubiquitination was much reduced with delayed PINK1 and DRP1 activation, which might be the result of impaired mitophagy initiation.^67^ In a fly HD model, over-expression of PINK1 corrected mitophagy and improved mitochondrial health.^9^ This suggests that the expression of high levels of HTT fragments itself promoted the induction of mitochondrial fission and resulted in slower, less dynamic mitophagy in response to stress. Chronic low-level impairment of mtDNA maintenance could underlie the accumulation of higher levels of pathogenic mtDNA mutations that we observed in two peripheral tissues in HD patients. In tissues with particularly high levels of somatic CAG repeat expansion and, subsequently, high levels of N-terminal mutant HTT fragments, such as the striatum,^68^ mtDNA quality control could be affected even more than in peripheral tissues, thus having more dramatic effects on mitochondrial health, in line with evidence from the N-terminal fragment R6/2 model.^15^

We have investigated peripheral tissues rather than brain tissue in a neurodegenerative disease. While this limits the conclusions that can be drawn for mitochondrial biology in the brain, our approach has several strengths. A major strength is that we used fresh tissue from human HD patients and age- and sex-matched controls. This avoided some of the technical challenges when examining post-mortem tissue. In addition, many studies examining HD use model systems that variously over-express different types of mutant HTT species. Over-expression of proteins in models may not entirely reflect what happens in humans carrying the *HTT* mutation with physiological expression levels. Peripheral tissues may be better suited as sources of biomarkers than brain tissue, so any insight into the effects of HD biology in accessible tissues can be very valuable, for instance as read-outs in clinical trials.^25^ It should also be noted that, while much greater than in previous studies, the number of skeletal muscle samples deep sequenced for mtDNA in the current study was still small compared with our lymphoblast work.^19^ More samples are needed to verify our observations and assess when mtDNA quality starts to deteriorate and how this relates to clinical onset and progression of HD (Fig. 5). The cell models we used differed substantially and thus did not allow a direct comparison of the influence of different HTT fragments on mitochondrial dynamics. We used primary fibroblasts with a CAG repeat expansion mutation length in the most common range for adult-onset HD. They express full-length human HTT and all possible HTT fragments and thus allow their impact on mitochondrial health to be studied. The HEK cell models solely harbour exon 1 of the mouse *Htt* gene with extremely long CAG repeats (>100). Such long CAG repeats either occur in individuals with childhood-onset HD or potentially result from somatic repeat expansion in cells (neurons) at a later disease stage in patients with adult-onset HD. We have also shown that even in cells that contain more exons of *HTT* and thus would be able to generate a spliced mRNA with these very long CAG repeat lengths, these cells almost exclusively produce *HTT1a* (the mRNA encoding for exon 1 HTT).^27^

Taken together, we showed mtDNA instability accumulating over the lifetime in HD patients, along with mechanistic evidence to indicate CAG repeat length dependently impaired mitochondrial quality control in response to stress. This is in line with our observation in lymphoblasts of HD patients, which demonstrated that CAG repeat length promotes expansion of pathogenic mtDNA mutations.^19^ In HD, an imbalance between levels of mitochondrial stress and the cellular ability to maintain mtDNA quality may not cause disease, unlike in some forms of Parkinson's disease with mutations in genes encoding PINK1 or parkin.^69^ However, the degree of mtDNA instability could modify primary HD pathogenesis, in line with genomic evidence derived from genome-wide association studies demonstrating a disease modifying effect of RRM2B sequence variation.^70,71^ RRMB2 encodes for a subunit of a ribonucleotide reductase contributing to mtDNA synthesis in non-proliferating cells with mutations causing mtDNA depletion.^72^ In addition to ongoing efforts to develop disease-modifying treatments directed towards the cause of HD, promoting mitochondrial health may be a complementary strategy. Improving mitochondrial quality control may be particularly valuable as it has been shown that promoting mitophagy can improve mitochondrial quality in model organisms and humans.^73^

## Supplementary Material

awae007_Supplementary_Data

## Data Availability

The muscle proteomics dataset is published alongside the MTM-HD data paper.^25^ MFA results are available in Supplementary Tables 1–4. The mtDNA mutations dataset is available in Supplementary Table 5. Code and scripts are available upon request from the corresponding author.
