# Supplementary material for: Huntington’s disease affects mitochondrial network dynamics predisposing to pathogenic mitochondrial DNA mutations
Source: Brain. 2024 Jan 9;147(6):2009–22. doi: 10.1093/brain/awae007 (PMC11512592; doi:10.1093/brain/awae007)
Supplement: awae007_Supplementary_Data [file awae007_supplementary_data.zip › brain-2023-01451-File014.pdf]

Huntington disease affects mitochondrial network dynamics predisposing to pathogenic mtDNA mutations

Andreas Neueder[1], Kerstin Kojer[1], Zhenglong Gu[2], Yiqin Wang[2], Tanja Hering[1], Sarah Tabrizi[3,4], Jan-Willem Taanman[3], and Michael Orth[1,5,6]

Blots relating to

Figure 3. The response to mitochondrial stress is diminished in HD fibroblasts and

Supplementary Figure 5. The response to mitochondrial stress in HD fibroblasts

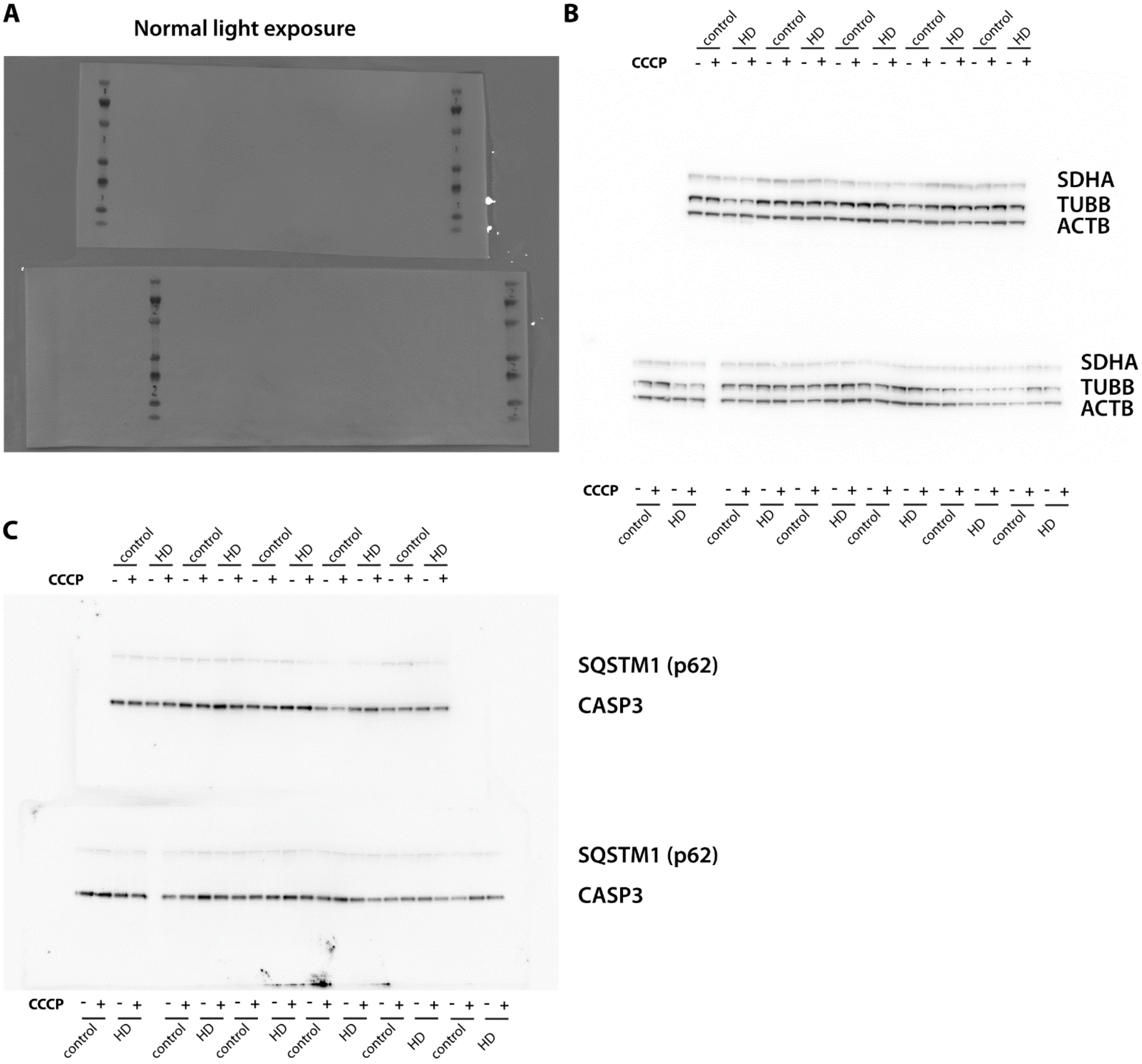

Primary human fibroblast lines 2 hours post CCCP treatment. The 4 samples on the lower left side of the size standard on the second blot, which were also run on the first blot, were used to normalise signals across the two blots. (**A**) Normal light exposure of the membranes. Western blot images for (**C**) p62 (SQSTM1), caspase 3 (CASP3), (**B**) succinate dehydrogenase complex, subunit A (SDHA), tubulin (TUBB) and actin (ACTB).

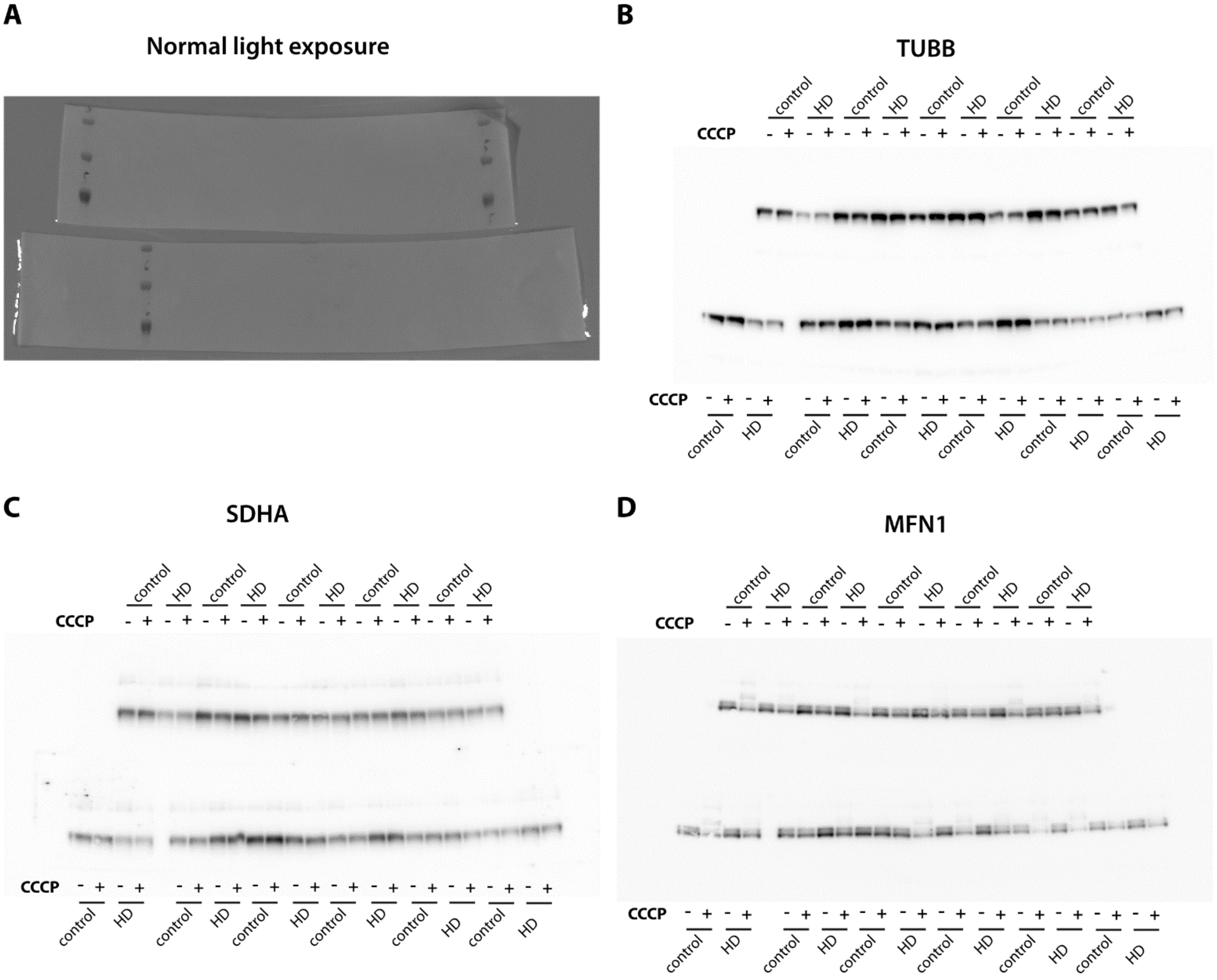

Primary human fibroblast lines 2 hours post CCCP treatment. The 4 samples on the lower left side of the size standard on the second blot, which were also run on the first blot, were used to normalise signals across the two blots. (**A**) Normal light exposure of the membranes. Western blot images for (**D**) mitofusin 1 (MFN1), (**C**) succinate dehydrogenase complex, subunit A (SDHA) and (**B**) tubulin (TUBB).

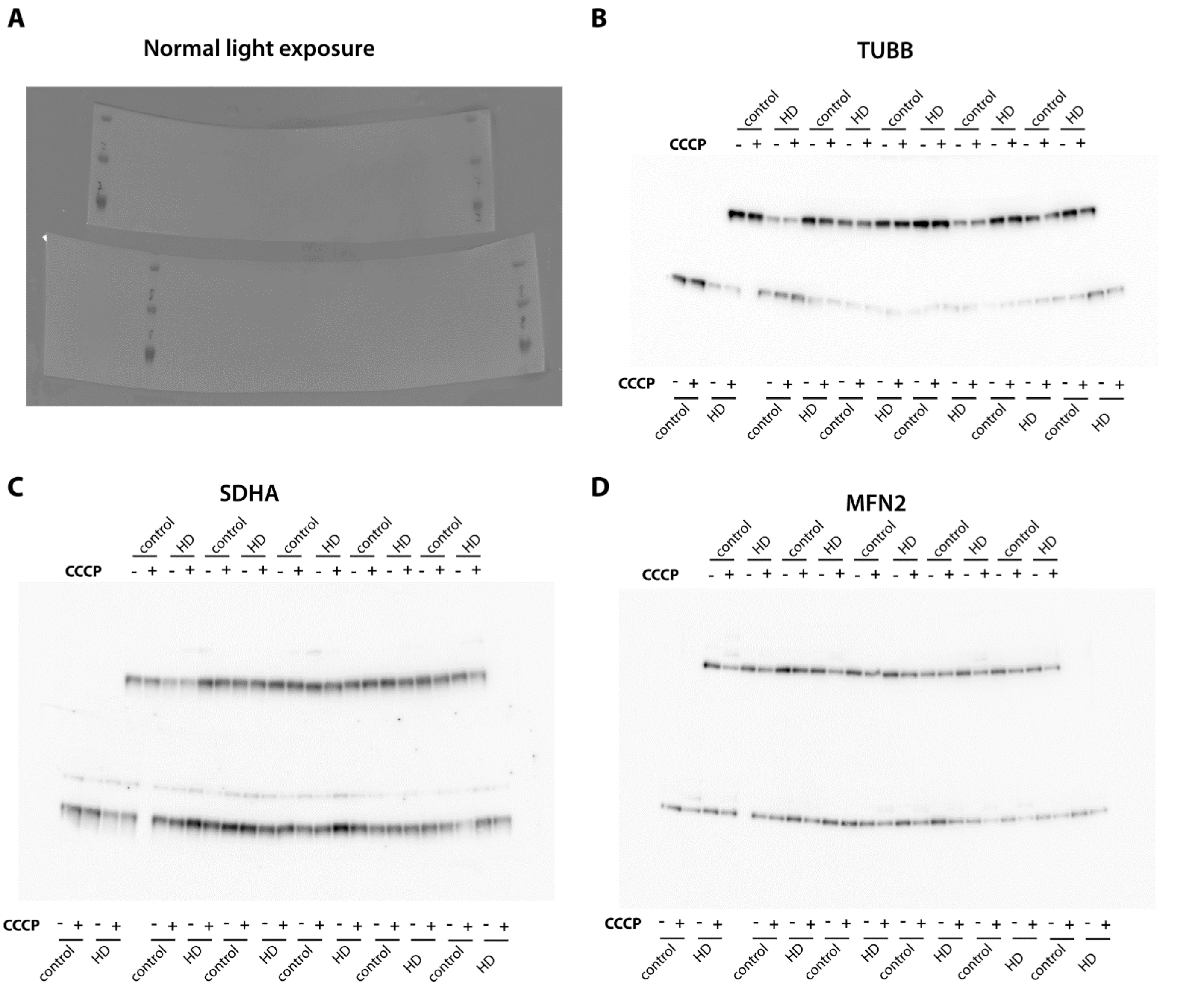

Primary human fibroblast lines 2 hours post CCCP treatment. The 4 samples on the lower left side of the size standard on the second blot, which were also run on the first blot, were used to normalise signals across the two blots. (**A**) Normal light exposure of the membranes. Western blot images for (**D**) mitofusin 2 (MFN2), (**C**) succinate dehydrogenase complex, subunit A (SDHA) and (**B**) tubulin (TUBB).

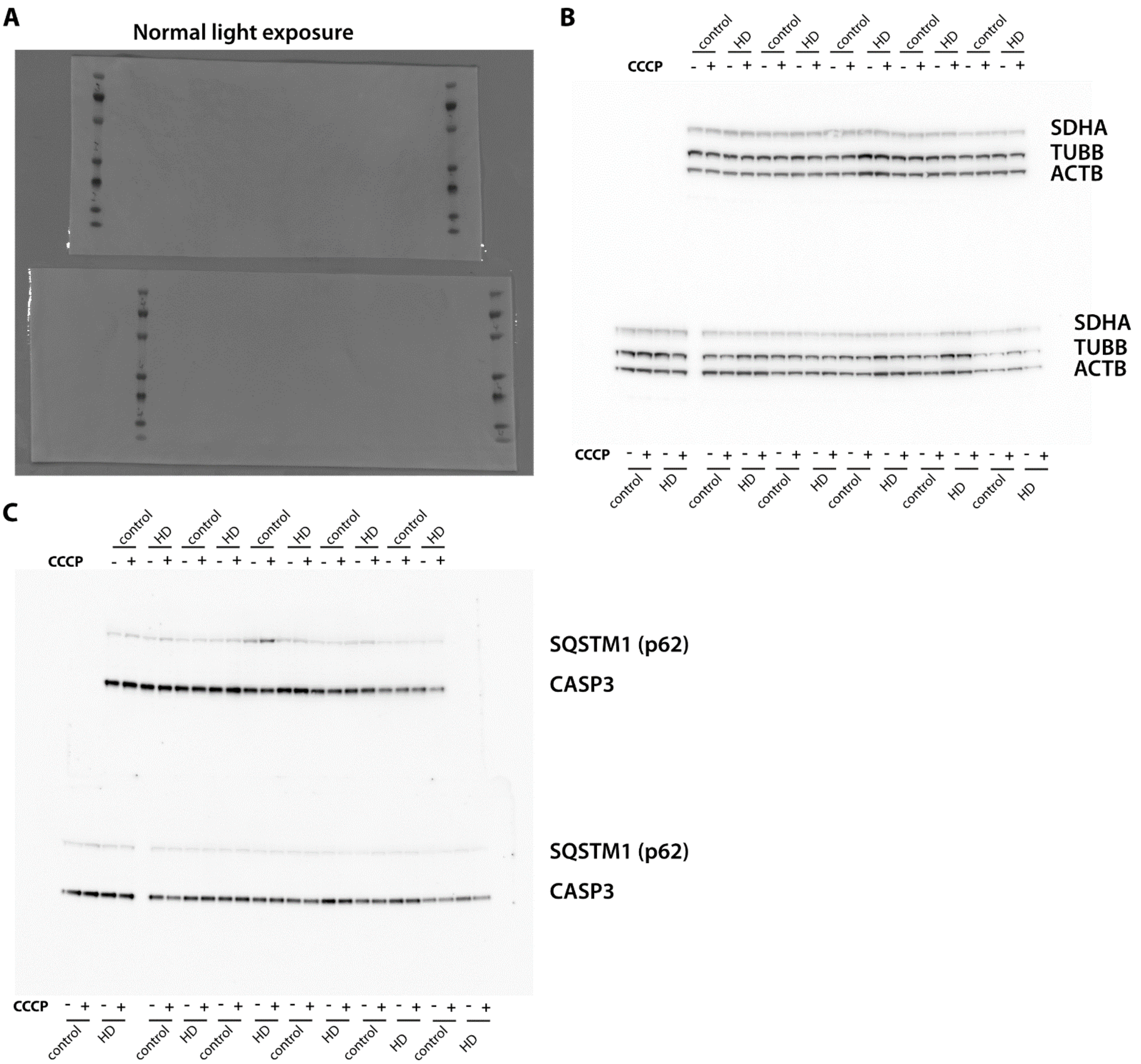

Primary human fibroblast lines 24 hours post CCCP treatment. The 4 samples on the lower left side of the size standard on the second blot, which were also run on the first blot, were used to normalise signals across the two blots. (**A**) Normal light exposure of the membranes. Western blot images for (**C**) p62 (SQSTM1), caspase 3 (CASP3), (**B**) succinate dehydrogenase complex, subunit A (SDHA), tubulin (TUBB) and actin (ACTB).

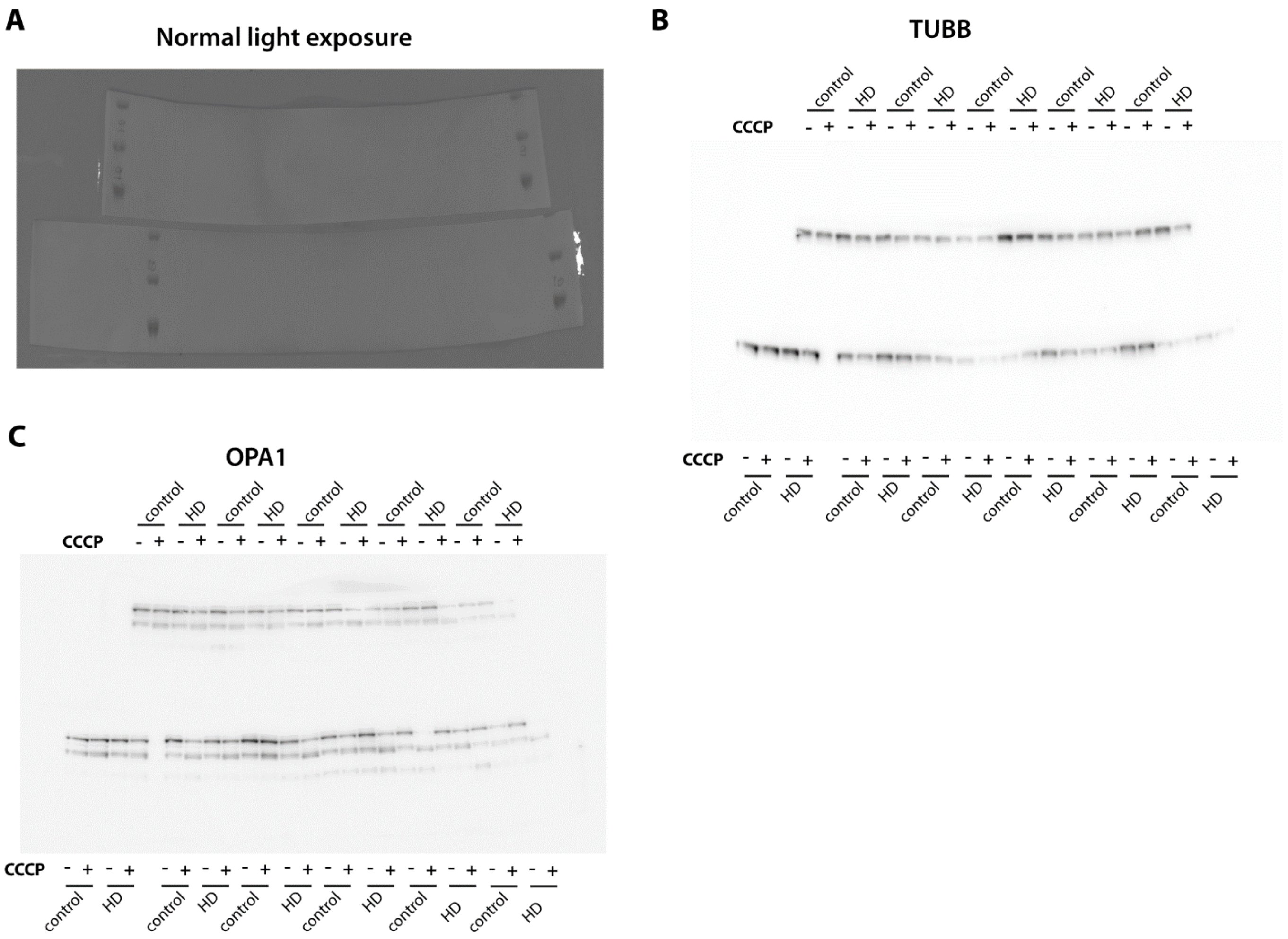

Primary human fibroblast lines 24 hours post CCCP treatment. The 4 samples on the lower left side of the size standard on the second blot, which were also run on the first blot, were used to normalise signals across the two blots. (**A**) Normal light exposure of the membranes. Western blot images for (**C**) OPA1 mitochondrial dynamin like GTPase (OPA1) isoforms and (**B**) tubulin (TUBB).

Blots relating to

Figure 4. Expression of exon 1 HTT further exacerbates the diminished response to mitochondrial stress and

Supplementary Figure 6. The response to mitochondrial stress in a HEK exon 1 HTT model,

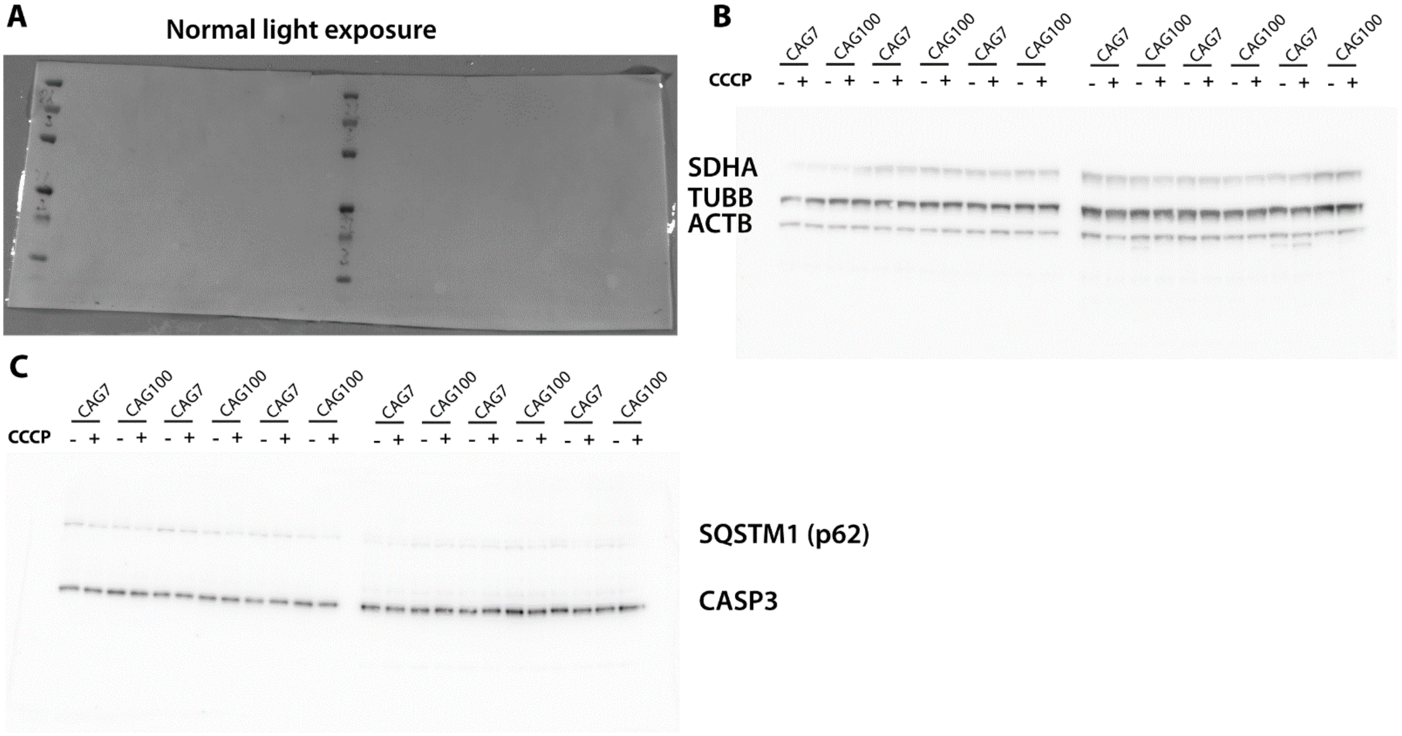

HEK293 cell line expressing exon 1 HTT with 7 (CAG_7) or 100 (CAG_{100}) CAG repeats 2 hours post CCCP treatment. (**A**) Normal light exposure of the membrane. Western blot images for (**C**) p62 (SQSTM1), caspase 3 (CASP3), (**B**) succinate dehydrogenase complex, subunit A (SDHA), tubulin (TUBB) and actin (ACTB).

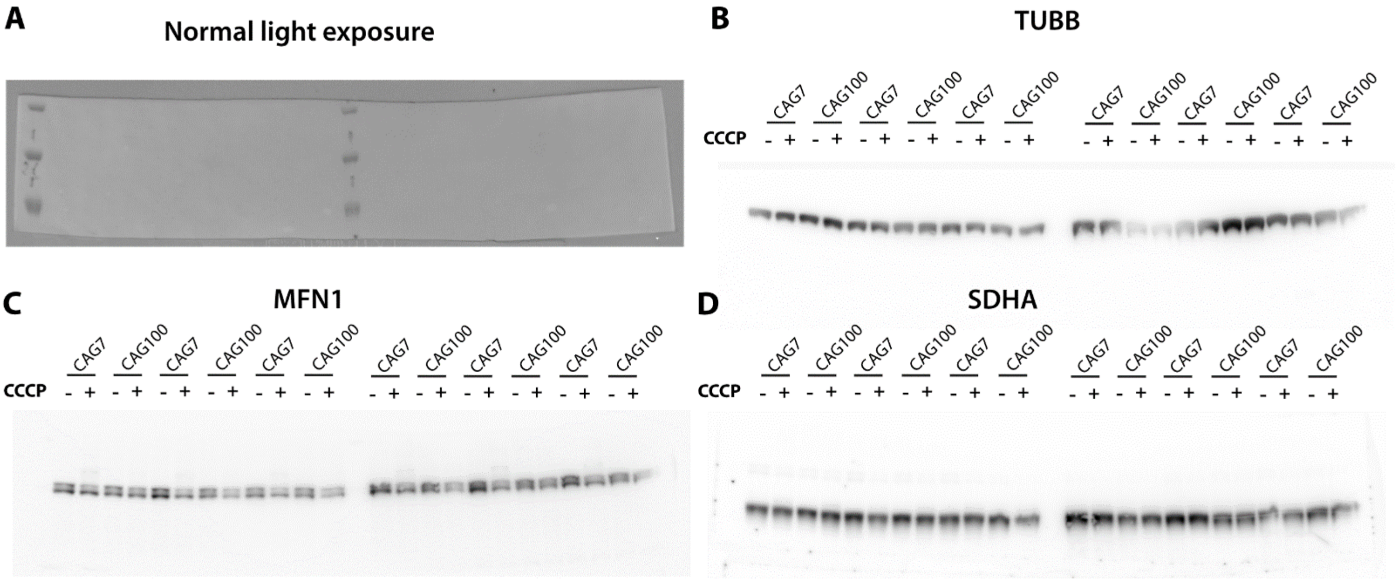

HEK293 cell line expressing exon 1 HTT with 7 (CAG_7) or 100 (CAG_{100}) CAG repeats 2 hours post CCCP treatment. Normal light exposure of the membrane. Western blot images for (**C**) mitofusin 1 (MFN1), (**B**) tubulin (TUBB) and (**D**) succinate dehydrogenase complex, subunit A (SDHA).

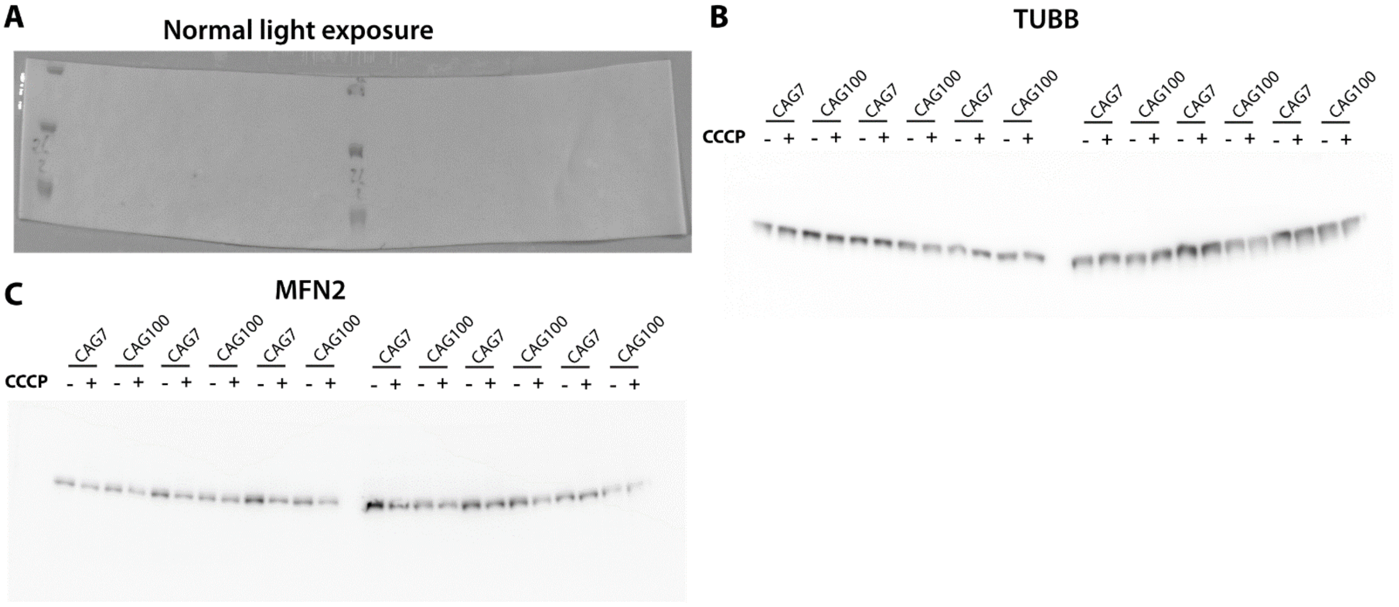

HEK293 cell line expressing exon 1 HTT with 7 (CAG_7) or 100 (CAG_{100}) CAG repeats 2 hours post CCCP treatment. Normal light exposure of the membrane. Western blot images for (**C**) mitofusin 2 (MFN2) and (**B**) tubulin (TUBB).

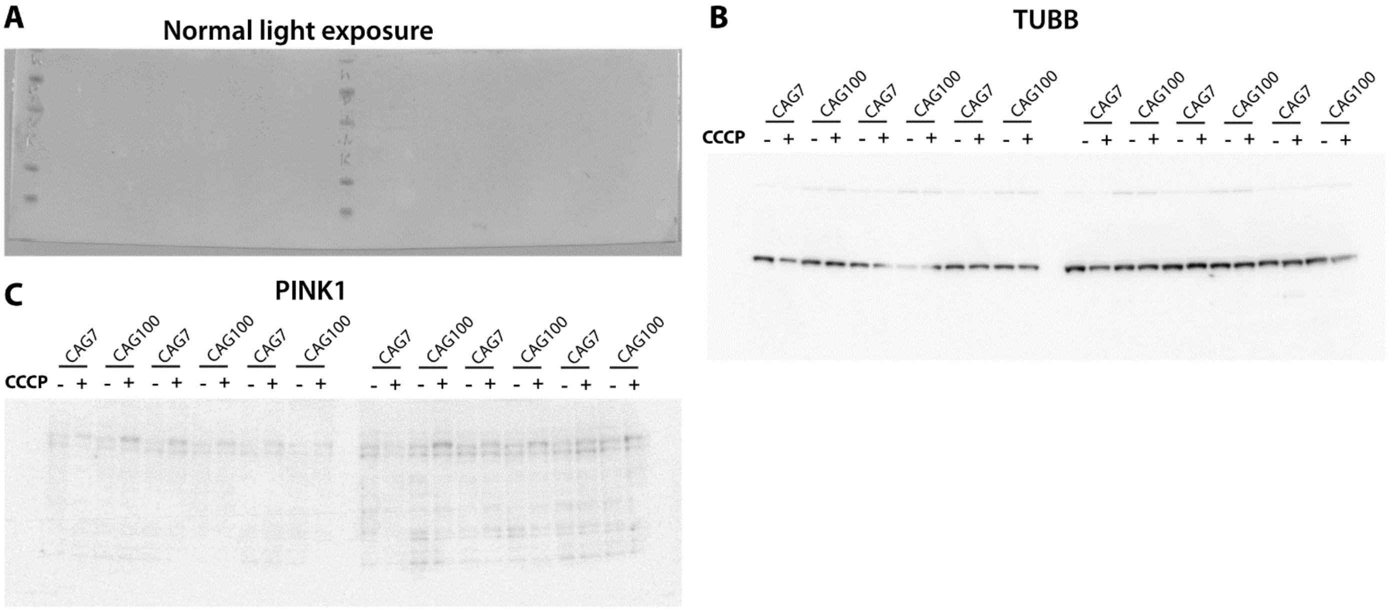

HEK293 cell line expressing exon 1 HTT with 7 (CAG_7) or 100 (CAG_{100}) CAG repeats 2 hours post CCCP treatment. Normal light exposure of the membrane. Western blot images for (**C**) PTEN induced kinase 1 (PINK1) and (**B**) tubulin (TUBB).

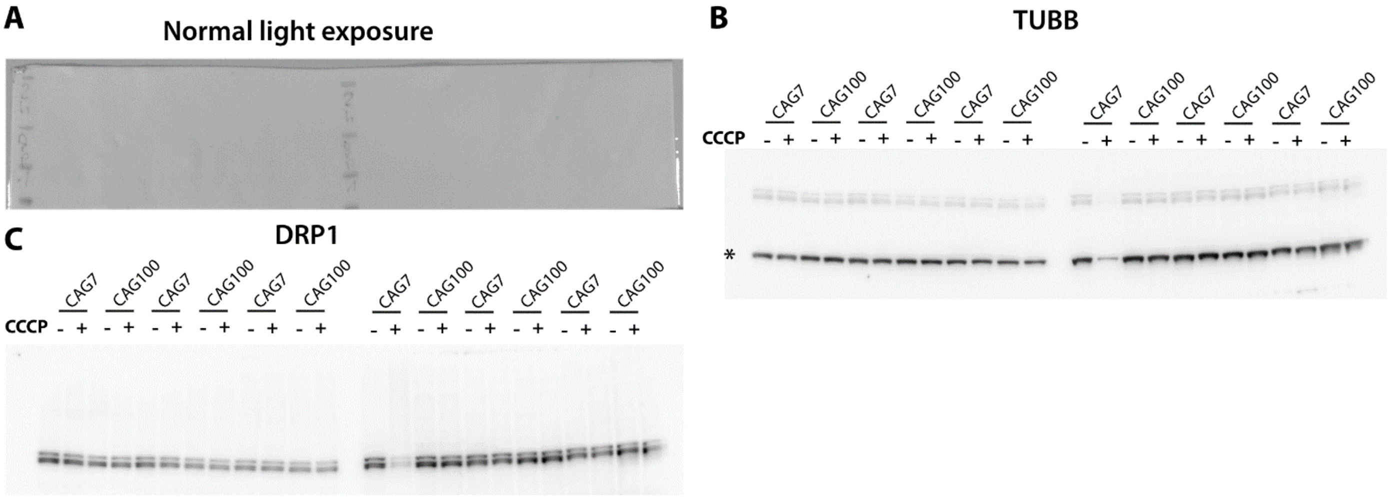

HEK293 cell line expressing exon 1 HTT with 7 (CAG_7) or 100 (CAG_{100}) CAG repeats 2 hours post CCCP treatment. Normal light exposure of the membrane. Western blot images for (**C**) dynamin-related protein 1 like (DNM1L/DRP1) and (**B**) tubulin (TUBB)*. For tubulin the bands of the previous exposure with anti-DRP1 antibody are still visible in the upper part of the membrane.

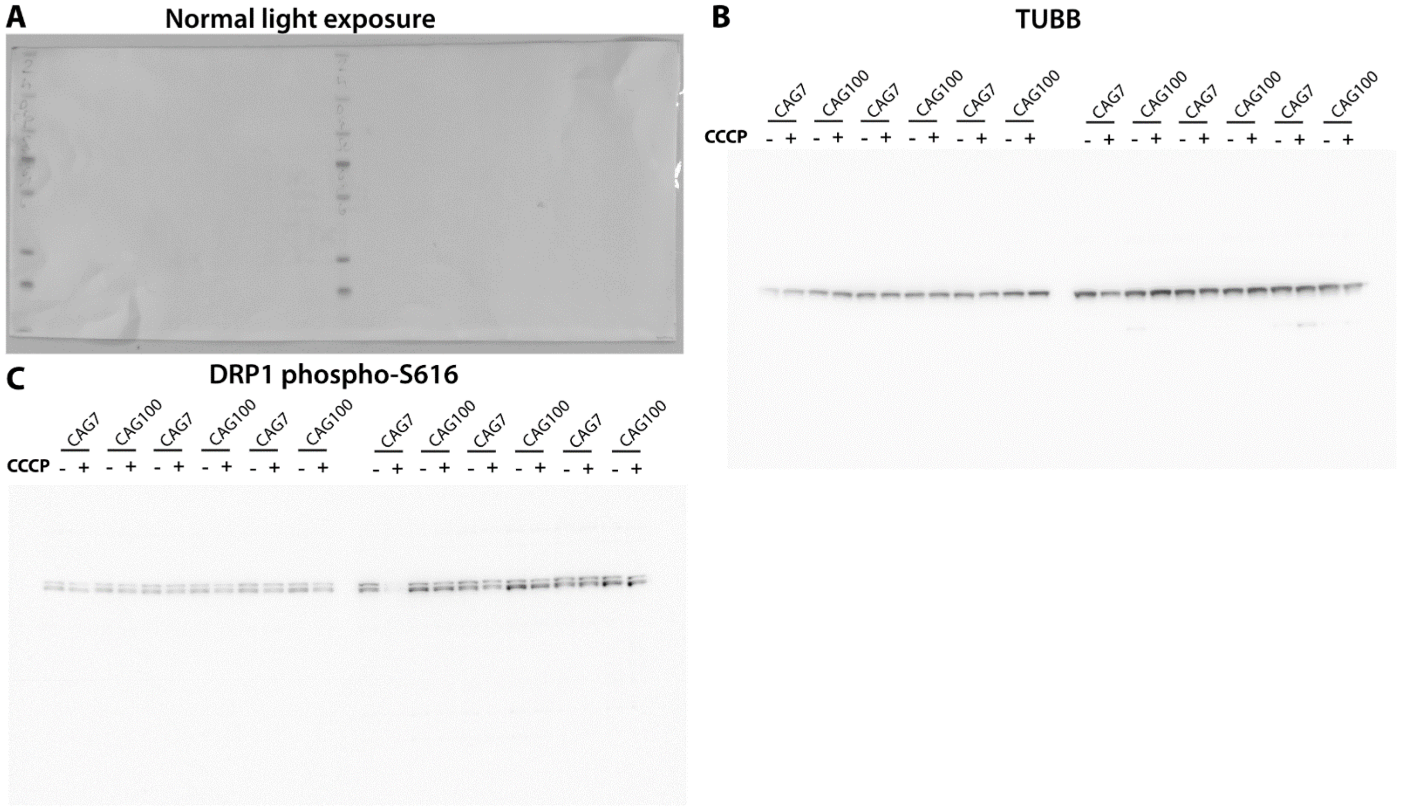

HEK293 cell line expressing exon 1 HTT with 7 (CAG_7) or 100 (CAG_{100}) CAG repeats 2 hours post CCCP treatment. Normal light exposure of the membrane. Western blot images for (**C**) phosphorylated (phosphor-S616) dynamin-related protein 1 like (DNM1L/DRP1) and (**B**) tubulin (TUBB).

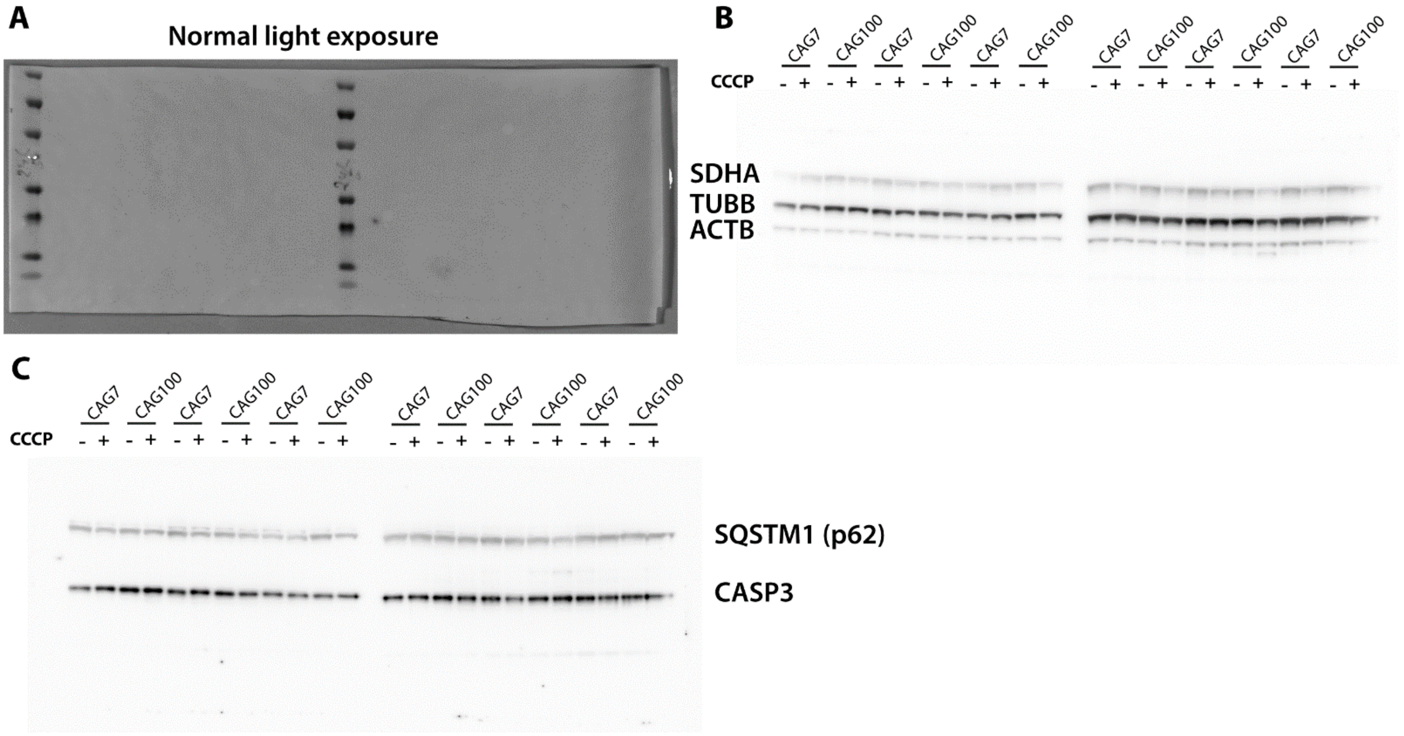

HEK293 cell line expressing exon 1 HTT with 7 (CAG_7) or 100 (CAG_{100}) CAG repeats 24 hours post CCCP treatment. Normal light exposure of the membrane. Western blot images for (**C**) p62 (SQSTM1), caspase 3 (CASP3), (**B**) succinate dehydrogenase complex, subunit A (SDHA), tubulin (TUBB) and actin (ACTB).

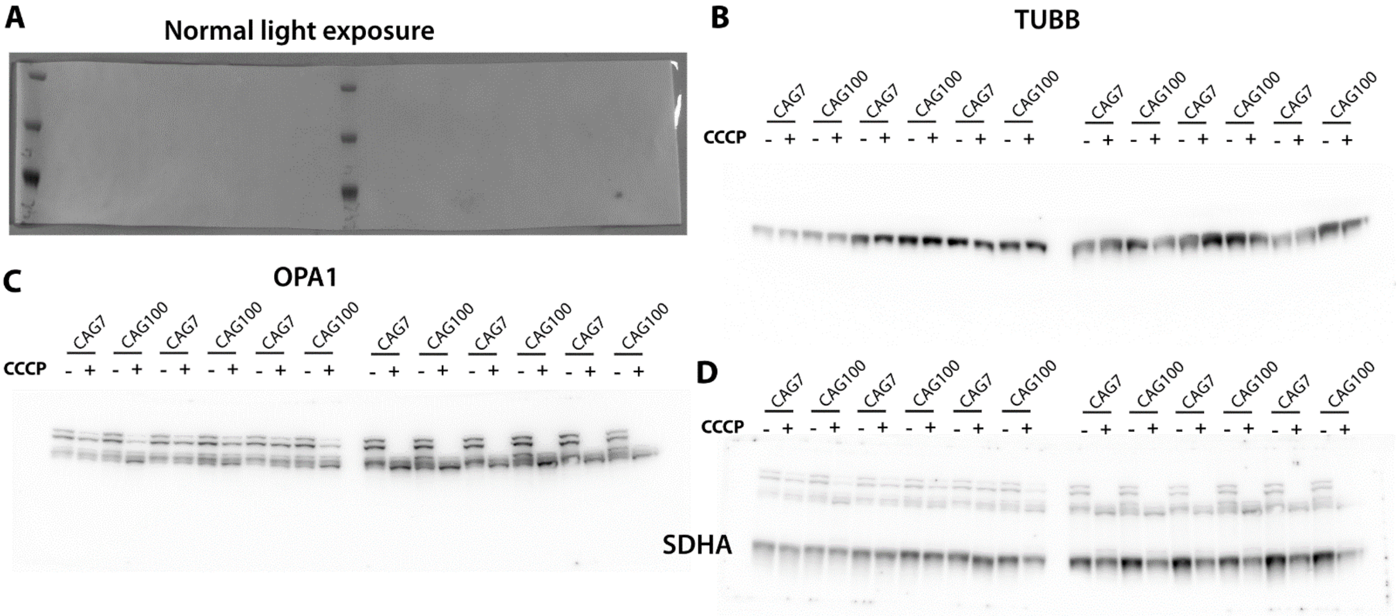

HEK293 cell line expressing exon 1 HTT with 7 (CAG_7) or 100 (CAG_{100}) CAG repeats 24 hours post CCCP treatment. Normal light exposure of the membrane. Western blot images for (**C**) OPA1 mitochondrial dynamin like GTPase (OPA1) isoforms, (**D**) succinate dehydrogenase complex, subunit A (SDHA) and (**B**) tubulin (TUBB). For SDHA the bands of the previous exposure with anti-OPA1 antibody are still visible in the upper part of the membrane.

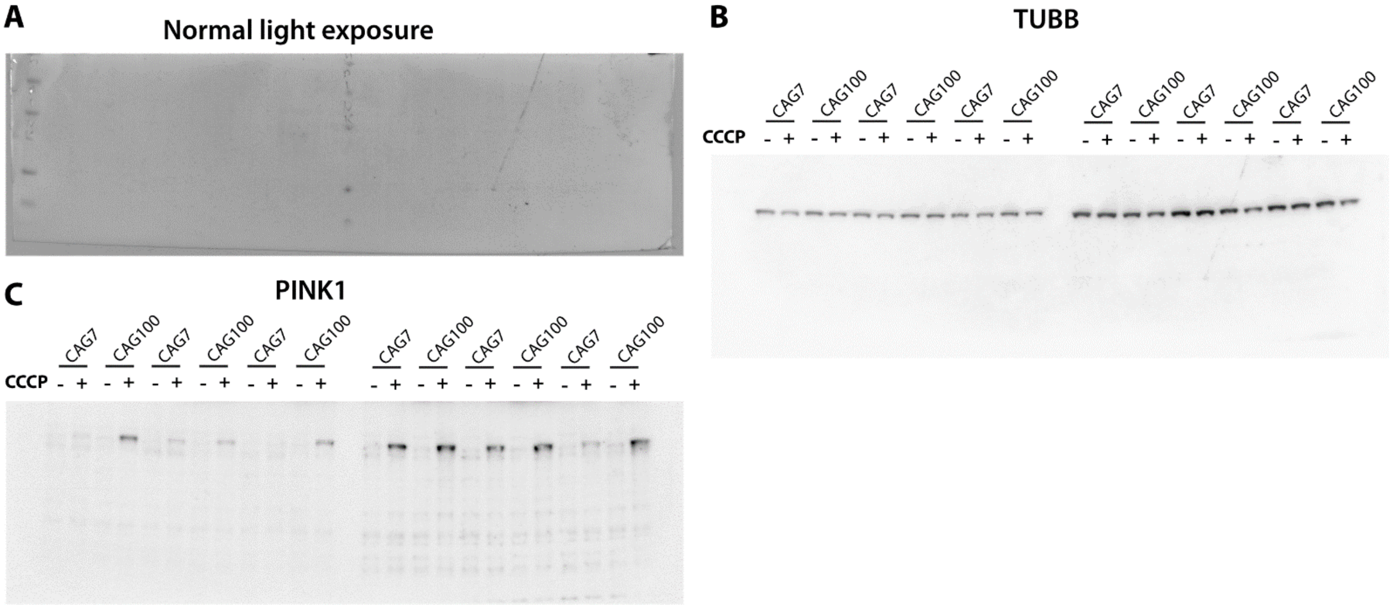

HEK293 cell line expressing exon 1 HTT with 7 (CAG_7) or 100 (CAG_{100}) CAG repeats 24 hours post CCCP treatment. Normal light exposure of the membrane. Western blot images for (**C**) PTEN induced kinase 1 (PINK1) and (**B**) tubulin (TUBB).

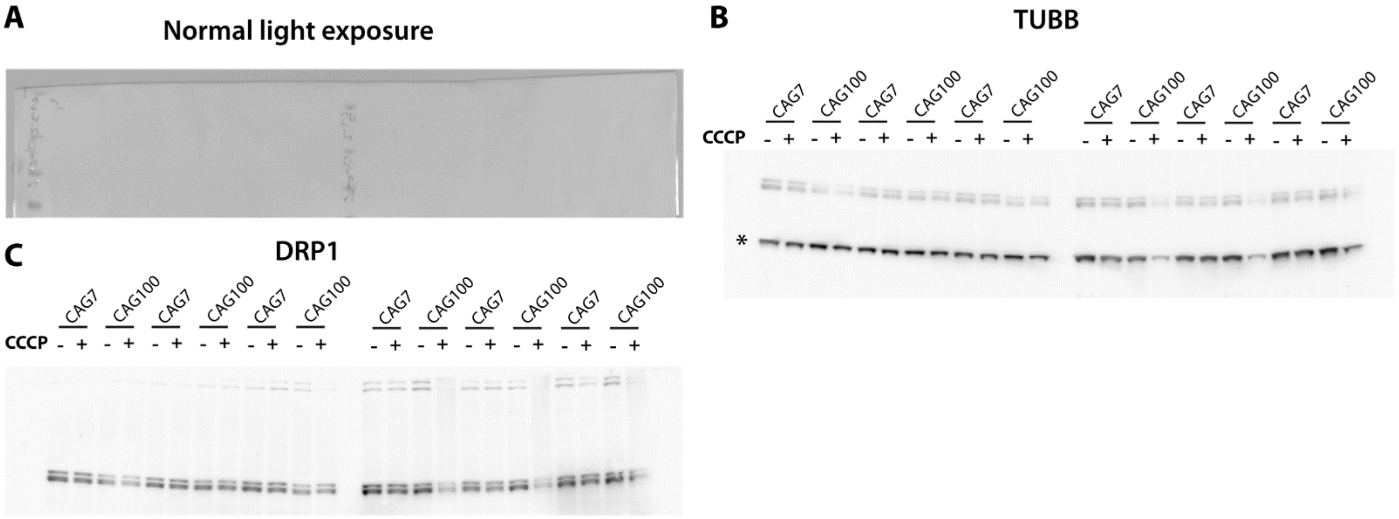

HEK293 cell line expressing exon 1 HTT with 7 (CAG_7) or 100 (CAG_{100}) CAG repeats 24 hours post CCCP treatment. Normal light exposure of the membrane. Western blot images for (**C**) dynamin-related protein 1 like (DNM1L/DRP1) and (**B**) tubulin (TUBB)*. For tubulin the bands of the previous exposure with anti-DRP1 antibody are still visible in the upper part of the membrane.

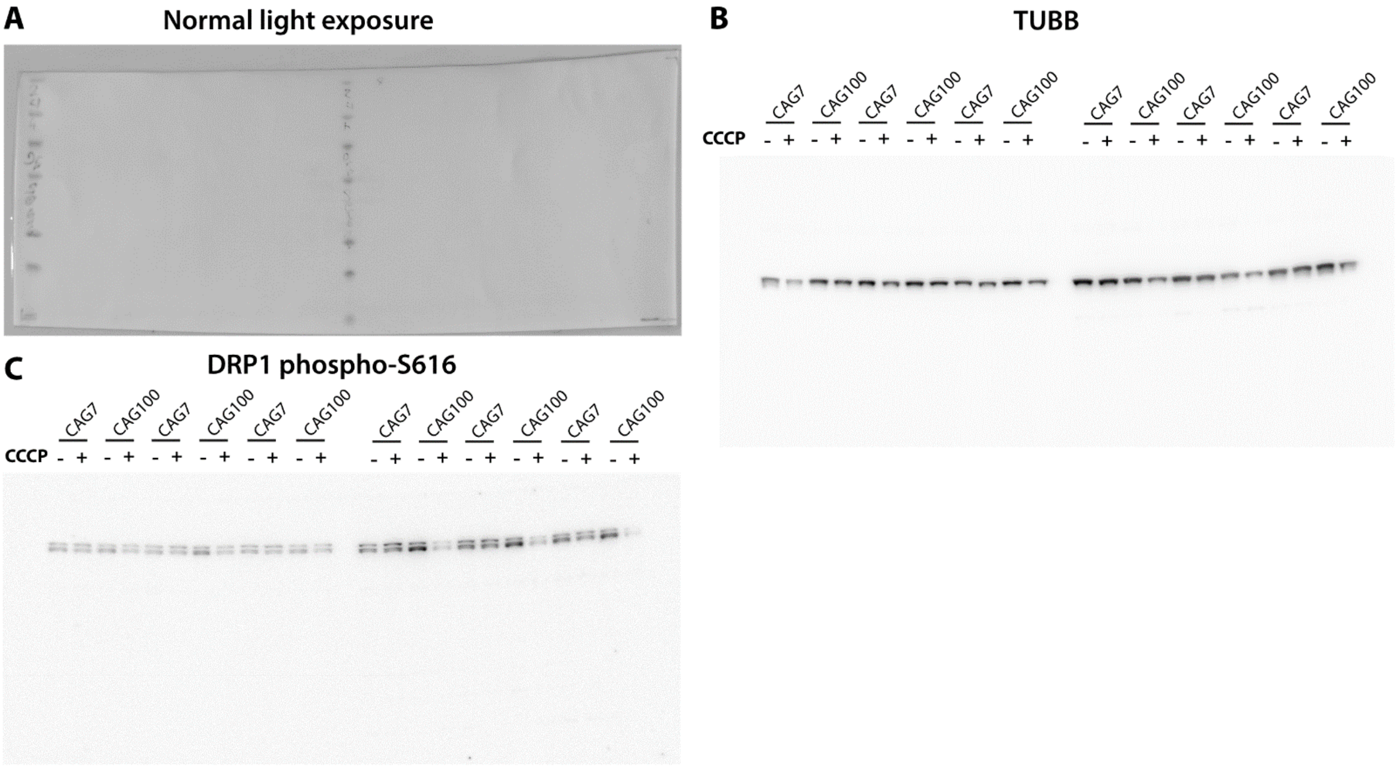

HEK293 cell line expressing exon 1 HTT with 7 (CAG_7) or 100 (CAG_{100}) CAG repeats 24 hours post CCCP treatment. Normal light exposure of the membrane. Western blot images for (**C**) phosphorylated (phosphor-S616) dynamin-related protein 1 like (DNM1L/DRP1) and (**B**) tubulin (TUBB).
