# Supplementary material for: Huntington’s disease affects mitochondrial network dynamics predisposing to pathogenic mitochondrial DNA mutations
Source: Brain. 2024 Jan 9;147(6):2009–22. doi: 10.1093/brain/awae007 (PMC11512592; doi:10.1093/brain/awae007)
Supplement: awae007_Supplementary_Data [file awae007_supplementary_data.zip › brain-2023-01451-File015.pdf]

Huntington disease affects mitochondrial network dynamics and predisposes to pathogenic mitochondrial DNA mutations

Andreas Neueder[1], Kerstin Kojer[1], Zhenglong Gu[2], Yiqin Wang[2], Tanja Hering[1], Sarah Tabrizi[3,4], Jan-Willem Taanman[3], and Michael Orth[1,5,6]

Supplementary Information

Supplementary Figures

Supplementary Fig. 1. Mitochondrial network analysis, related to Fig. 1.
Supplementary Fig. 2. Mitochondrial network analysis in human fibroblasts, related to Fig. 2.
Supplementary Fig. 3. Mitochondrial network analysis in human myoblasts, related to Fig. 2.
Supplementary Fig. 4. Mitochondrial network analysis in a HEK293 cell line expressing exon 1 HTT, related to Fig. 2.
Supplementary Fig. 5. The response to mitochondrial stress in HD fibroblasts, related to Fig. 3.
Supplementary Fig. 6. The response to mitochondrial stress in a HEK exon 1 HTT model, related to Fig. 4.

Supplementary Tables

Supplementary Table 1: MFA results – association of MFA dimensions against categorical variables (Separate Excel file)
Supplementary Table 2: MFA results – quantitative variables (Separate Excel file)
Supplementary Table 3: MFA results – quantitative supplementary variables (Separate Excel file)
Supplementary Table 4: MFA results – groups (Separate Excel file)
Supplementary Table 5: mtDNA mutations (Separate Excel file)
Supplementary Table 6: Mitochondrial mass and activity data, related to Fig. 1

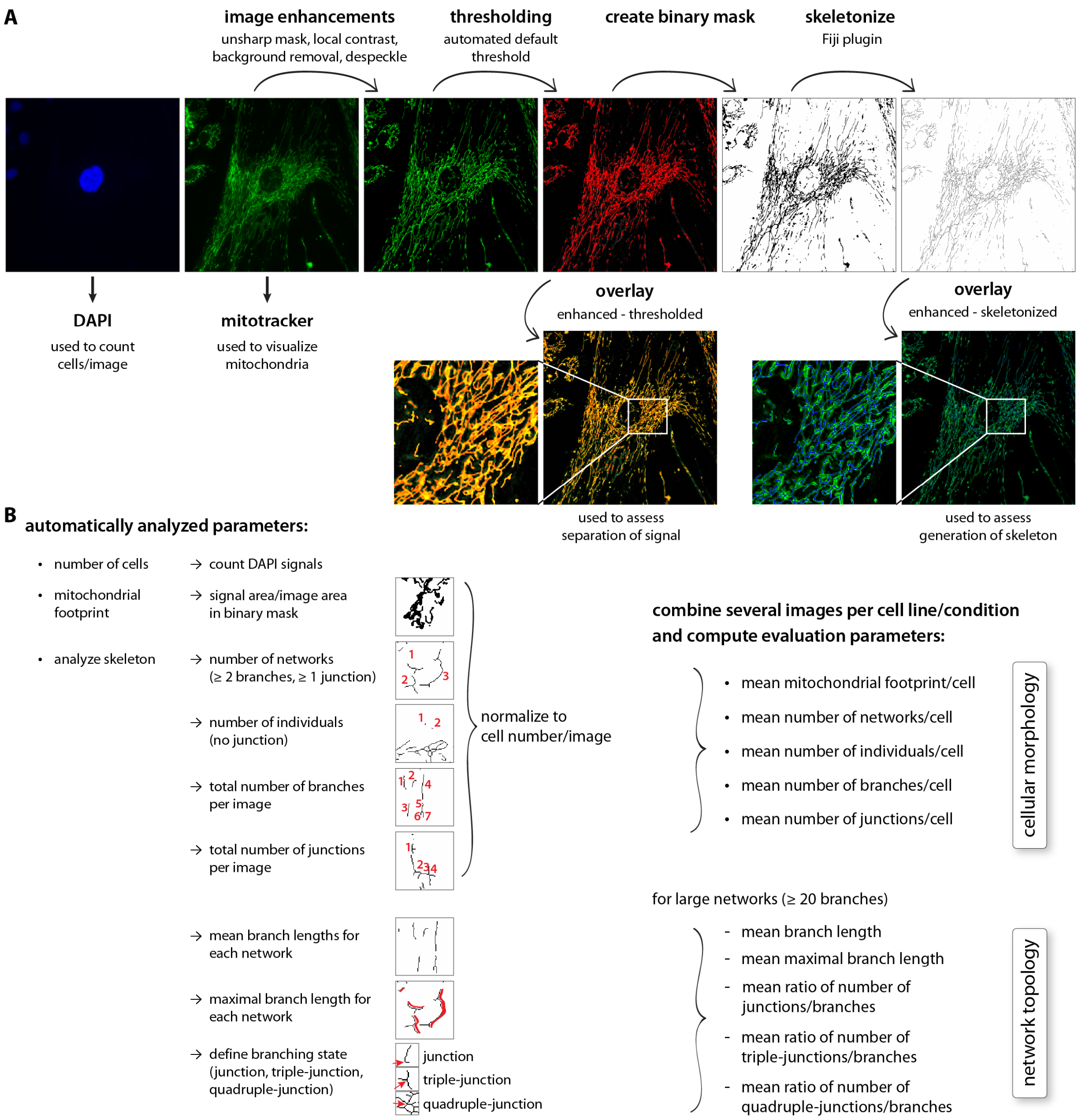

Supplementary Figure 1 Mitochondrial network analysis. (**A**) Workflow of the mitochondrial network analysis. DAPI (nuclei) signals were used to count cell per image. Mitotracker (mitochondrial networks) signal was enhanced using several filters in Fiji (see also Methods). Signal was separated from background and binarized. The resulting image was skeletonized and analyzed using the Fiji plugin. Overlay images of enhanced original and signal separated images were used to assess the thresholding/separation performance. Overlay images of enhanced original and skeletonized images were used to assess the generation of the network data/skeletons. (**B**) The workflow was scripted as a macro and run unsupervised on all images. Raw data generated were as shown in the figure. We defined a minimal mitochondrial network as having equal to or greater than two branches and one junction. Otherwise signal was counted as an individual, i.e. having no junction. Parameters describing the mitochondrial

cellular morphology were normalized to the cell number per image as these depend on the analyzed number of cells. Parameters describing the network topology were only calculated for large networks. Variance in smaller networks was high, we therefore used only networks with equal to or greater than 20 branches for the topology analysis. This figure is linked to Fig. 2.

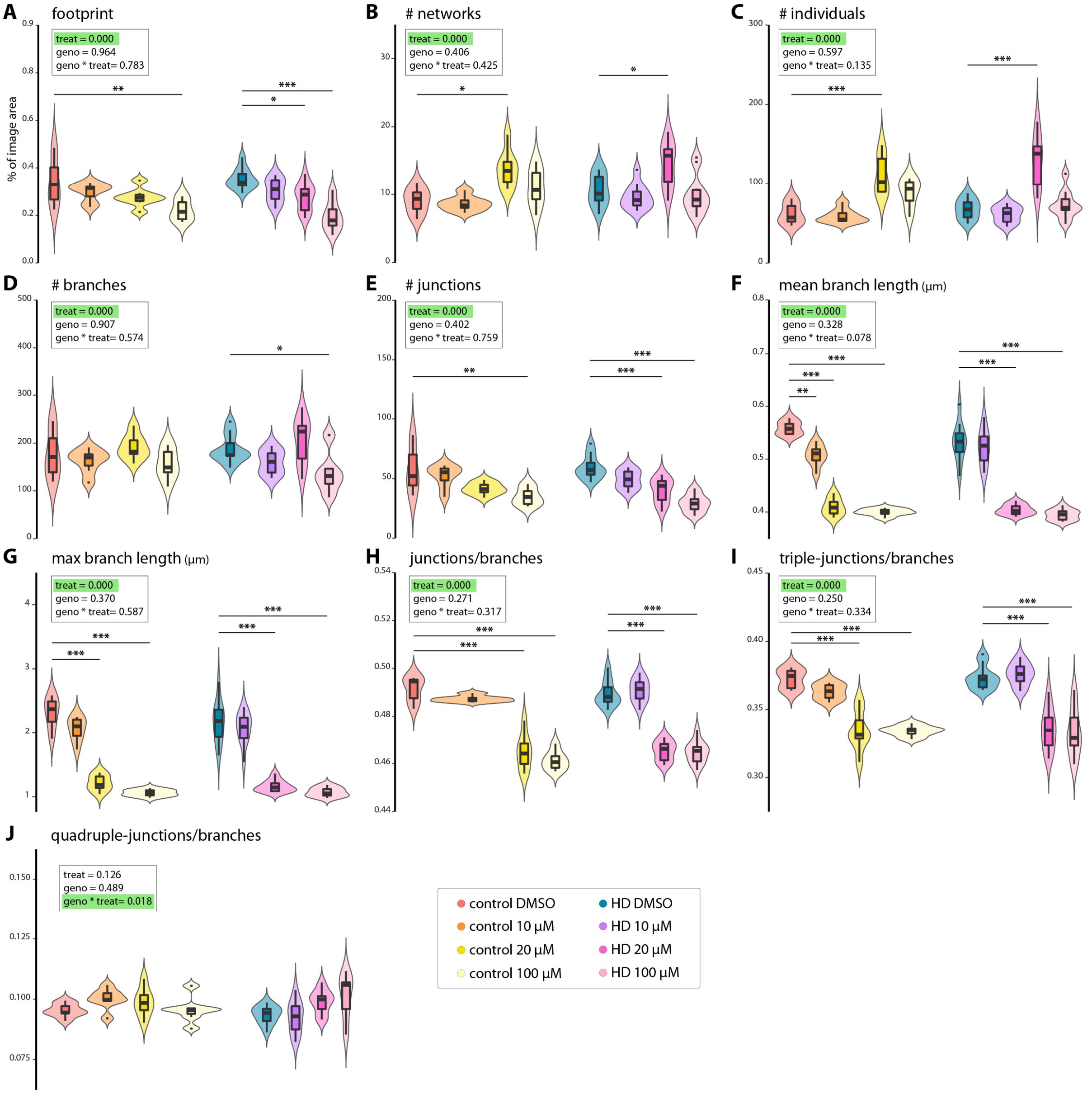

Supplementary Figure 2 Mitochondrial network analysis in human fibroblasts. (A - J) Cells were either treated with DMSO, or mitochondrial stress was induced for 24 hours by treatment with 10, 20 or 100 µM CCCP. Data are violin plots together with inlayed box plots. Violin plots show the full distribution of the data by the colored density area that represents all measured data. Statistics is a two-way ANOVA with Tukey post-hoc test. The main effects for treatment (treat, CCCP vs. DMSO) and genotype (geno, HD vs. control) including the interaction of treatment and genotype (geno * treat) are shown. Treatment: *$p < 0.05$; **$p < 0.01$; ***$p < 0.001$. Control $n = 7$; HD $n = 11$. This figure is linked to Fig. 2.

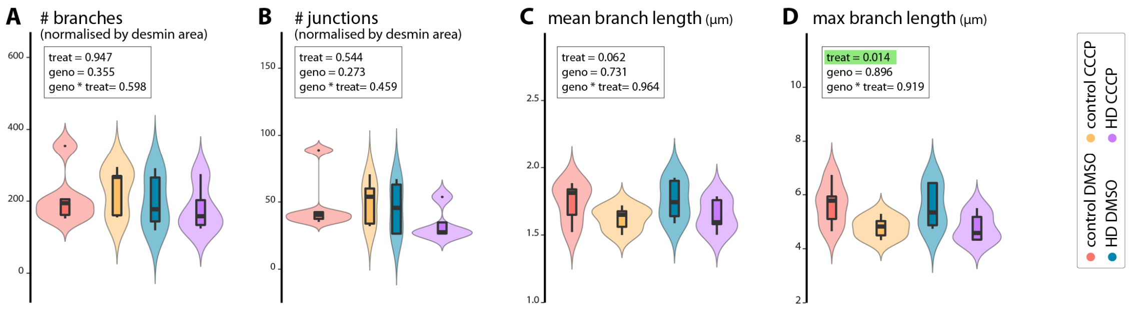

Supplementary Figure 3 Mitochondrial network analysis in human myoblasts. (**A** - **D**) Cells were either treated with DMSO, or mitochondrial stress was induced for 24 hours by treatment with 10 µM CCCP. Only desmin positive cells were analyzed and data was normalized to the desmin area (cell area). Data are violin plots together with inlayed box plots. Violin plots show the full distribution of the data by the colored density area that represents all measured data. Statistics is a two-way ANOVA with Tukey post-hoc test. The main effects for treatment (treat, CCCP vs. DMSO) and genotype (geno, HD vs. control) including the interaction of treatment and genotype (geno * treat) are shown. Controls n = 5; HD n = 5. This figure is linked to Fig. 2.

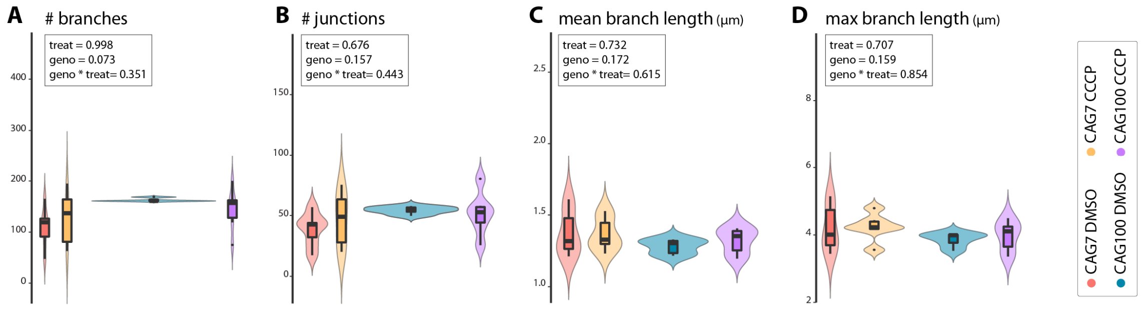

Supplementary Figure 4 Mitochondrial network analysis in a HEK293 cell line expressing exon 1 HTT. (**A** - **D**) Cells were either treated with DMSO, or mitochondrial stress was induced for 24 hours by treatment with 10 µM CCCP. Data are violin plots together with inlayed box plots. Violin plots show the full distribution of the data by the colored density area that represents all measured data. Statistics is a two-way ANOVA with Tukey post-hoc test. The main effects for treatment (treat, CCCP vs. DMSO) and genotype (geno, CAG100 vs. CAG7) including the interaction of treatment and genotype (geno * treat) are shown. CAG7/CAG100 n = 6/6 of independent experiments. This figure is linked to Fig. 2.

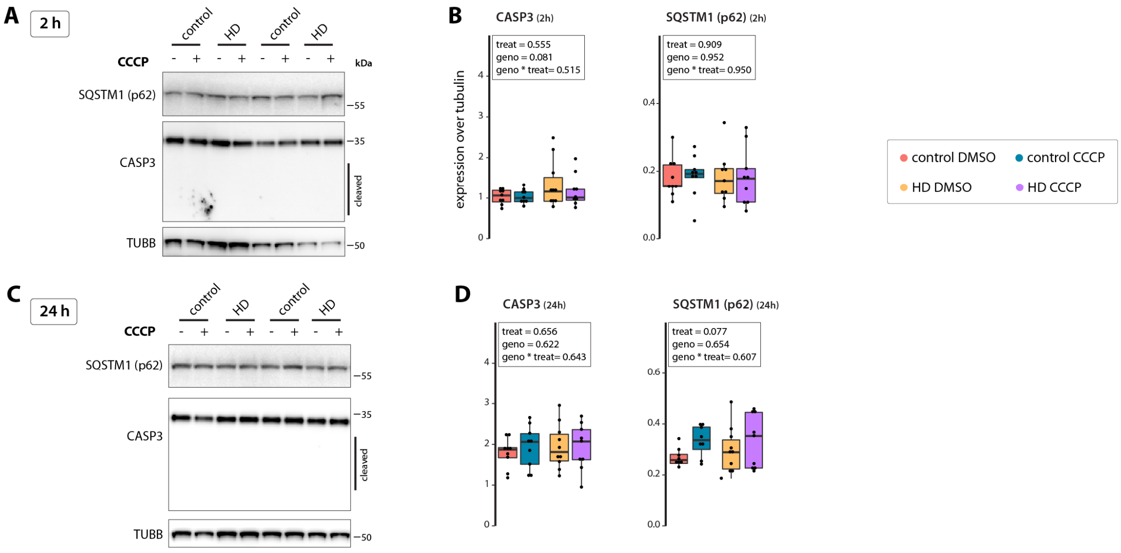

Supplementary Figure 5 The response to mitochondrial stress in HD fibroblasts. Protein expression analysis of human fibroblasts after induction of mitochondrial stress by treatment with 10 µM CCCP for 2 hours (**A** and **B**) or 24 hours (**C** and **D**). (**A**) Western blot images for caspase 3 (CASP3), sequestosome 1 (SQSTM1/p62) and tubulin (TUBB). (**B**) Quantification of protein expression levels shown in (**A**), normalized to tubulin. (**C**) Western blot images for caspase 3 (CASP3), sequestosome 1 (SQSTM1/p62) and tubulin (TUBB). (**D**) Quantification of protein expression levels shown in (**C**), normalized to tubulin. (B and D) Data are box plots. *N* = 10 for all groups. Statistics is a two-way ANOVA with Tukey *post-hoc* test. The main effects for treatment (treat, 10 µM CCCP vs. DMSO) and genotype (geno, HD vs. control) including the interaction of treatment and genotype (geno * treat) are shown. This figure is linked to Fig. 3.

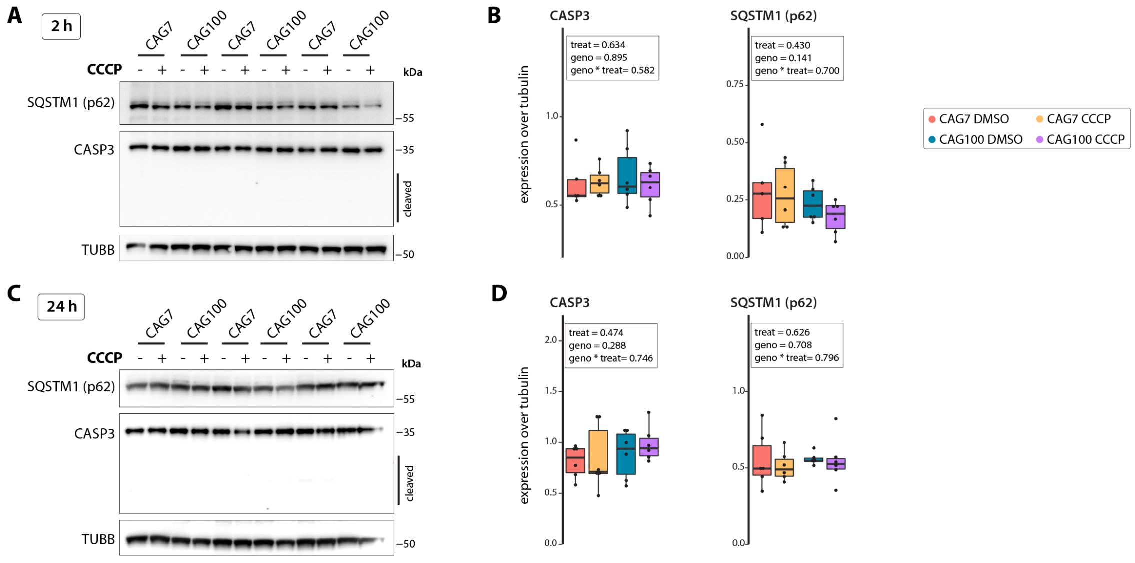

Supplementary Figure 6 The response to mitochondrial stress in a HEK exon 1 HTT model. Protein expression analysis of a HEK exon 1 HTT model after induction of mitochondrial stress by treatment with 10 µM CCCP for 2 hours (**A** and **B**) or 24 hours (**C** and **D**). (**A**) Western blot images for caspase 3 (CASP3), sequestosome 1 (SQSTM1/p62) and tubulin (TUBB). (**B**) Quantification of protein expression levels shown in (A), normalized to tubulin. (**C**) Western blot images for caspase 3 (CASP3), sequestosome 1 (SQSTM1/p62) and tubulin (TUBB). (**D**) Quantification of protein expression levels shown in (**C**), normalized to tubulin. (**B** and **D**) Data are box plots. $N = 6$ independent experiments for all groups. Statistics is a two-way ANOVA with Tukey *post-hoc* test. The main effects for treatment (treat, 10 µM CCCP vs. DMSO) and genotype (geno, CAG100 vs. CAG7) including the interaction of treatment and genotype (geno * treat) are shown. This figure is linked to Fig. 4.

Supplementary Table 6 Mitochondrial mass and activity data

	HTT protein	mtDNA copies	CS activity[a]	ACO activity[a]	Complex I activity[b]	Complex II/III activity[b]	Complex IV activity[b]
**control**	1.0 (0.105)	14490 (4551)	1.0 (0.170)	1.0 (0.243)	1.0 (0.115)	1.0 (0.218)	1.0 (0.145)
**pre-HD**	1.693 (0.117)	11759 (3076)	1.078 (0.202)	0.840 (0.181)	1.044 (0.095)	0.865 (0.169)	1.115 (0.155)
**early-HD**	1.768 (0.084)	10380 (2524)	1.037 (0.156)	0.627 (0.134)	0.936 (0.107)	0.582 (0.111)	0.949 (0.174)
**ANOVA control vs. pre-HD**[c]	**< 0.001**	n.s.	n.s.	n.s.	n.s.	n.s.	n.s.
**ANOVA control vs. early-HD**[c]	**< 0.001**	n.s.	n.s.	n.s.	n.s.	n.s.	n.s.
**ANOVA control vs. HD**[c]	**< 0.001**	n.s.	n.s.	n.s.	n.s.	n.s.	n.s.

All data were normalised to the respective control group with the exception of mtDNA copies. Average with standard error in brackets is given.

[a]Citrate synthase (CS) and aconitase (ACO) activity were normalised to CS and ACO protein levels, respectively. Activity is nmol/min per mg protein.

[b]Complex I, II/III and IV activity were normalised to total protein content (BCA). Activity is nmol/min per mg protein.

[c]Multivariate ANOVA with Bonferroni *post hoc* with 'site of sampling' and 'sex' as co-variates. n.s. = not significant ($p > 0.05$)
